# Identification of an Immune-Related Signature for Predicting Prognosis in Patients With Pancreatic Ductal Adenocarcinoma

**DOI:** 10.3389/fonc.2020.618215

**Published:** 2021-02-24

**Authors:** Weijia Wang, Liang Yan, Xiaoya Guan, Bin Dong, Min Zhao, Jianhui Wu, Xiuyun Tian, Chunyi Hao

**Affiliations:** ^1^ Key Laboratory of Carcinogenesis and Translational Research, Ministry of Education, Department of Hepato-Pancreato-Biliary Surgery, Peking University Cancer Hospital & Institute, Beijing, China; ^2^ Key Laboratory of Carcinogenesis and Translational Research, Ministry of Education, Central Laboratory, Peking University Cancer Hospital & Institute, Beijing, China; ^3^ Key Laboratory of Carcinogenesis and Translational Research, Ministry of Education, Department of Pathology, Peking University Cancer Hospital & Institute, Beijing, China

**Keywords:** immune-related genes, immune signature, pancreatic ductal adenocarcinoma, prognosis, tumor microenvironment

## Abstract

**Purpose:**

Pancreatic ductal adenocarcinoma (PDAC) is one of the highest fatality rate cancers with poor survival rates. The tumor microenvironment (TME) is vital for tumor immune responses, leading to resistance to chemotherapy and poor prognosis of PDAC patients. This study aimed to provide a comprehensive evaluation of the immune genes and microenvironment in PDAC that might help in predicting prognosis and guiding clinical treatments.

**Methods:**

We developed a prognosis-associated immune signature (i.e., PAIS) based on immune-associated genes to predict the overall survival of patients with PDAC. The clinical significance and immune landscapes of the signature were comprehensively analyzed.

**Results:**

Owing to gene expression profiles from TCGA database, functional enrichment analysis revealed a significant difference in the immune response between PDAC and normal pancreas. Using transcriptome data analysis of a training set, we identified an immune signature represented by 5 genes (ESR2, IDO1, IL20RB, PPP3CA, and PLAU) related to the overall survival of patients with PDAC, significantly. This training set was well-validated in a test set. Our results indicated a clear association between a high-risk score and a very poor prognosis. Stratification analysis and multivariate Cox regression analysis revealed that PAIS was an important prognostic factor. We also found that the risk score was positively correlated with the inflammatory response, antigen-presenting process, and expression level of some immunosuppressive checkpoint molecules (e.g., CD73, PD-L1, CD80, and B7-H3). These results suggested that high-risk patients had a suppressed immune response. However, they could respond better to chemotherapy. In addition, PAIS was positively correlated with the infiltration of M2 macrophages in PDAC.

**Conclusions:**

This study highlighted the relationship between the immune response and prognosis in PDAC and developed a clinically feasible signature that might serve as a powerful prognostic tool and help further optimize the cancer therapy paradigm.

## Introduction

Pancreatic ductal adenocarcinoma (PDAC) is the fourth leading contributing factor of cancer -related death worldwide, exhibiting an extremely poor 5-year survival (less than 9%) (1). By 2030, PDAC is estimated to rise to the second leading cause of cancer mortality (2) owing to its aggressive malignancy and limited therapeutic options. Although various systemic therapies have been developed, their overall efficacy and survival benefits remain limited, probably owing to the abundance of the stromal content of the tumor (3, 4). Therefore, it is important to develop new treatments for patients with pancreatic cancer.

Immunotherapies, which can fight tumors by activating the immune system, have achieved great success in recent years. Although immune checkpoint inhibitors (ICIs) have shown demonstrated clinical affection in the management of several malignancies (5–8) to date, they have been generally ineffective in PDAC (9). However, some clinical trials have shown that therapies targeting the tumor microenvironment (TME) might ameliorate the prognosis of patients with PDAC. Among a retrospective research, patients in the ICIs plus chemotherapy set showed a better prognosis than those in the single chemotherapy set (10). Compounds or therapeutic approaches that target the P450 cytochrome (11) or immune mediators, including legumain (12) and Toll-like receptors (13), were also demonstrated to decrease the impact of the TME on tumor progression. In a phase II adjuvant study, GVAX, which is composed of allogenic irradiated pancreatic cancer cells, can generate granulocyte-macrophage colony stimulating factor (GM-CSF), result in the expansion of CD8^+^ T-cells and help patients to achieve longer survival times (14–16). Another study showed that ruxolitinib, a JAK/STAT inhibitor, combined with capecitabine was reported to improve the survival of patients with metastatic PDAC who insensitive to chemotherapy with gemcitabine (17).

The TME of PDAC is unique, consisting of cancer cells, stromal cells, and extracellular components and might promote tumor evasion, conferring resistance to therapeutic agents and immune therapies (18). Recent studies have demonstrated that TME is vital for PDAC progression (19). Tumor-infiltrating immune cells (TIICs), migration from peripheral blood or exist in PDAC, could also implicate positive or negative regulation of tumor growth and progression according to the type and their functional interactions (20). Up-regulating of tumor supporting cells, for example, tumor-associated macrophages (TAMs), while down-regulating of the killers of tumor cells, such as CD8^+^ T-cells and natural killer (NK) cells, is necessary for immunosuppressive microenvironment in PDAC (21).

These findings have supported the importance of TME in PDAC, but the molecular mechanisms behind it, particularly those relevant to the effects of immune-related genes (IRGs), remain unclear. With the available large-scale public gene expression datasets, researchers can identify potential biomarkers for tumor surveillance much faster and more accurately (22, 23). Recently, the prognostic value of IRGs-in patients with nonsquamous non-small cell lung cancer has been developed (24). Thus, we examined whether IRGs could form a prognostic-associated gene signature in PDAC

In this study, we executed gene set enrichment analysis (GSEA) in gene expression profiles from the TCGA and GTEx datasets. We found significant differences in the immune response that could be used to distinguish PDAC from a healthy pancreas. We then developed a prognosis-associated immune signature (PAIS) in 163 PDAC samples obtained from the TCGA dataset. This signature was validated using an independent set of 95 tumor samples from the ICGC dataset. We also analyzed the enrichment pathway and transcription factor (TF) regulation network of survival-associated IRGs. We comprehensively analyzed the clinical significance, immune checkpoint profiles, and immune cell infiltration of the PAIS. The results of our analysis might provide an opportunity to further optimize the paradigm of cancer therapy and effectively develop a prognostic tool for PDAC.

## Materials and Methods

### Public Gene Expression Datasets

Gene expression profiles of patients with PDAC and healthy donors were acquired from the TCGA database (https://tcga-data.nci.nih.gov/tcga), GTEx database (https://www.gtexportal.org/home/index.html), and ICGC Data Portal (https://dcc.icgc.org/), respectively. In total, 163 samples of PDAC tissues from TCGA, 95 samples of PDAC tissues from ICGC-AU, and 165 samples of normal pancreatic tissues from the GTEx dataset were included in this study. The transcriptome data were log 2 transformed and standardized across patients using a quantile normalized method. All corresponding characteristics and clinical outcomes of enrolled patients were publicly obtainable. The patient information from the TCGA and ICGC databases are presented in **Table S1**.

### Analysis of Differentially Expressed Immune-Related Genes

We extracted the list of IRGs using the publicly accessible Immunology Database and Analysis Portal (ImmPort) database (https://www.immport.org) (25). This database can provide a set of IRGs participating in the immune activity. We used the DEseq2 package to screen for differentially expressed IRGs between PDAC samples from TCGA and normal pancreas tissue samples (26). Differential gene analysis was subjected to all transcriptional data, we chose the false discovery rate (FDR) < 0.01 and a log2 |fold change| > 1 as the cutoff values.

### Enrichment Analysis of Immune-Related Genes

Kyoto Encyclopedia of Genes and Genomes (KEGG) and gene set enrichment analysis (GSEA) are two commonly used enrichment analysis methods We extracted GSEA between PDAC sample and normal pancreas, and high- and low risk PDAC samples for better understanding of entirety changes (27). On the other hand, we chose KEGG analysis of the differentially expressed IRGs and to conducted the exploration of molecular mechanisms to further exploring different immune-related pathways between different expressed IRGs.

### Construction and Validation of the Prognosis-Associated Immune Signature

Considering the generally poor prognosis of most cases of PDAC, we chose the overall survival (OS) for the PAIS building, deriving all data from the TCGA’s Pan-Cancer Atlas (28). TCGA was the train set with 163 patients while the ICGC was the test set with 165 patients. We performed univariate Cox proportional hazards regression modeling to evaluate their prognostic value for OS in the training set (29). Based on minimal criteria, the least absolute shrinkage and selection operator (LASSO) Cox proportional hazards regression model was used to select the genes with the largest predictive values (30). Next, we applied a multivariate Cox proportional hazards regression model to determine the target genes forming a PAIS for prognostication. To calculate the PAIS value of each patient, we designed a formula that included weighting the normalized expression value of the target genes by their respective coefficients. In the formula, the expression values of the target genes were normalized with a mean value = 0 and a standard deviation (SD) = 1 to obtain a uniform cutoff value to assign patients into low-risk or high-risk sets (31). The prognostic power of the novel PAIS for OS was evaluated in two different cohorts using receiver operating characteristic curve (ROC) and Kaplan-Meier survival analyses. We also performed univariate and multivariate Cox regression analyses to assess whether PAIS was an independent prognostic risk factor.

### Analysis of Differentially Expressed Transcription Factors

Transcription factors (TFs) can control the intensity of gene expression directly. Hence, exploration of the status of TFs which may have the potential ability to regulate these survival-relevant IRGs is necessary. Cistrome Cancer (http://cistrome.org/CistromeCancer/) is a public database that includes 318 TFs, thus making it valuable for cancer biology research (32).

### CIBERSORT and Assessment of Tumor-Infiltrating Immune Cells

CIBERSORT(https://cibersort.stanford.edu/) is one of the methods for calculating the infiltration of immune cells. It used a deconvolution algorithm according to the standardized gene expression, thus could be able to predict the proportion of different immune cells in samples (33–35). Here, we used CIBERSORT to transform the gene expression of PDAC to an abundance of TIICs in PDAC tissue samples. Selected the filtered data (75 cases, P<0.05) for further analysis. We calculated the correlation between immune cells and clinical characteristics and the association between the infiltration of immune cells and PAIS.

### Clinical Drug Response Prediction

We performed the prediction analysis of Paclitaxel, one of the commonly used drugs for chemotherapy in PDAC, using the “pRRophetic” R package. Our analysis used ridge regression to estimate the half-maximal inhibitory concentration (IC50) of each sample. The prediction accuracy of the model was tested using 10-fold cross-validation based on the Genomics of Drug Sensitivity in Cancer (GDSC) (https://www.cancerrxgene.org/) training set. All parameters in the program were set based on default values and duplicate gene expression was identified as the mean value (36).

### Statistical Analysis

GraphPad Prism software (version 8.0) and R software (version 3.6.2) were used to perform the statistical analyses. The survival curves for OS from the Kaplan-Meier survival analysis were compared using log-rank tests. Chi-square and Mann-Whitney U tests were applied for between-set comparisons. All reported P-values were two-tailed. For all analyses, P-values of less than 0.001(***), 0.01(**), or 0.05(*) were considered statistically significant.

## Results

### Relationships of Immune Phenotype Between Pancreatic Ductal Adenocarcinomaand Normal Pancreas

**Figure 1:**
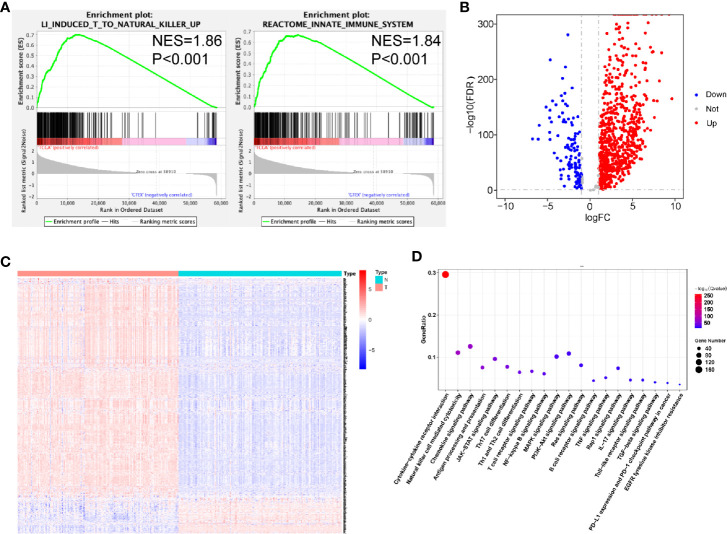
Relationships between pancreatic ductal adenocarcinoma (PDAC) and normal pancreatic tissues. **(A)** Significant enrichment of immune-related pathways in tissues from patients with PDAC, compared with normal pancreatic tissues. NES, normalized enrichment score. **(B)** Volcano plot of 871 metabolism-related genes differentially expressed in PDAC and normal pancreatic samples. **(C)** Heatmap showing the expression of IRGs in each sample. **(D)** Functional analysis of 838 metabolism-related genes.

To examine the distinct features of biological processes in PDAC compared with normal pancreas, we analyzed the gene expression between 163 PDAC samples obtained from TCGA and 165 samples from GTEx. Accordingly, we performed GSEA of PDAC and normal pancreatic tissue samples. The GSEA results revealed that PDAC was strongly correlated with positive regulation of the immune response, including the induction of T-cells to natural killer cells (NES = 1.86, P < 0.001) and activation of the innate immune system (NES = 1.84, P < 0.001) (**Figure 1A**). To further investigate the association between PDAC and immune phenotypes, we chose the 871 immune-related genes extracted from the ImmPort database for further calculation. We found that among these genes, 837 were differentially expressed between PDAC samples and normal pancreas (**Figures 1B, C**, **Table S2**). Next, we used KEGG enrichment analysis to identify immune-related pathways associated with these 837 significant genes. These genes were identified to be involved in cytokine-cytokine receptor interaction, Th17 cell differentiation, Th1 and Th2 cell differentiation, as well as in the expression of PD-L1 and the PD-1 checkpoint pathway (**Figure 1D**).

In addition, we observed that the JAK-STAT, NF-κB, PI3K-AKT, and MAPK signaling pathways were enriched (**Figure 1D**).

### Development of a Prognosis-Associated Immune Signature for Pancreatic Ductal Adenocarcinoma in the Training Cohort

**Figure 2:**
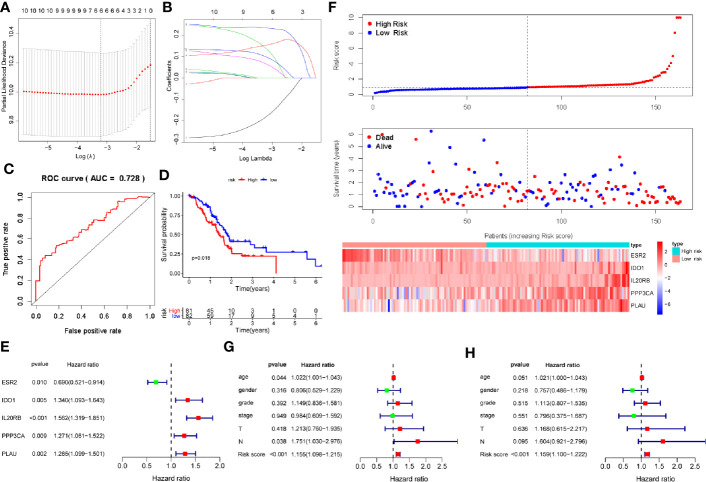
Development of prognosis-associated immune signature (PAIS) for pancreatic ductal adenocarcinoma (PDAC) in the training cohort. **(A, B)** The least absolute shrinkage and selection operator (LASSO) Cox proportional hazards regression model identified 10 genes most related to overall survival (OS). **(C)** ROC curve analysis of the PAIS for OS. **(D)** Kaplan-Meier survival curves of OS for patients with PDAC. **(E)** Prognostic values of five selected genes using multivariate Cox proportional hazards regression analysis. **(F)** Heatmap of five-gene expression profiles, PAIS distributions, and live status of each patient in the high- and low-risk sets. **(G, H)** Univariate **(G)** and multivariate **(H)** regression analyses of the associations between PAIS and clinical variables for the predictive value of OS.

We found significant differences in the immune response and immune-related signaling pathway between PDAC tissues and normal pancreas. Therefore, we sought to develop a PAIS to improve the prognostic prediction for PDAC. As the 5-year survival rate of patients with PDAC is extremely poor, we chose the postoperation overall survival (OS) as the clinical outcome. To this end, we performed univariate Cox proportional hazards regression analysis to identify IRGs correlated with OS. All the prognosis-associated IRGs analyzed by Kaplan-Meier and Univariable Cox regression were shown in **Tables S3** and **S4**, respectively. Based on a P-value < 0.01, 10 immune-related genes were identified as prognostic genes for OS. Subsequently, we used LASSO Cox proportional hazards regression modeling to select genes with the greatest predictive values. Based on the minimum criteria, 6 genes were selected (**Figures 2A, B**). We then performed multivariate Cox regression analysis to further generate a PAIS for prognostication, and accordingly a novel prognostic signature consisting of 5 genes (ESR2, IDO1, IL20RB, PPP3CA, and PLAU) was built (**Figure 2D**). Subsequently, we generated the PAIS model for each patient using the formula: PAIS = −0.298 × normalized expression value of ESR2 + 0.259 × normalized expression value of IDO1 + 0.298 × normalized expression value of IL20RB + 0.172 × normalized expression value of PPP3CA + 0.184 × normalized expression value of PLAU (**Figure 2E**). PDAC patients were assigned into low- and high-risk sets based on the optimal cutoff value (0.931), which was obtained from the median value of the PAIS in the training set (**Figure 2F**).

To evaluate the predictive power of the novel PAIS, we obtained the area under the curve (AUC) values of the ROC and performed the Kaplan-Meier survival analysis. Our results revealed that the AUC value at 1-year OS was 0.728 (**Figure 2C**). Patients in the high-risk set were shown to have a significantly worse OS than those in the low-risk set (P = 0.018; **Figure 2D**). Next, to examine whether the PAIS was an independent risk factor for OS in patients with PDAC, we performed univariate and multivariate Cox regression analyses of the training set. The results of Cox regression analysis indicated that both PAIS and lymph node metastasis were predictor factors (PAIS: P < 0.001; age: P = 0.044; lymph node metastasis: P = 0.038; **Figure 2G**). However, after adjusting for clinicopathological factors, including age, gender, grade, stage, T–staging, and lymph node metastasis, our results indicated that PAIS was a major independent predictor factor of OS (P < 0.001; **Figure 2H**). In addition, PAIS was observed to be significantly higher in patients with advanced stage (**Table 1**).

**Table 1:** Relationships between the expressions of the immune-related genes (IRGs) and risk score and the clinicopathological factors in pancreatic adenocarcinoma: t(P).

gene	Age(<65/≥65)	Gender(female/male)	Grade(G1-G2/G3-G4)	Stage(stage I - II/stage III-IV)	T(T1-T2/T3-T4)	N(N0/N1)
ESR2	−0.408(0.684)	−0.408(0.684)	2.317(0.022)	2.713(0.022)	−0.659(0.513)	0.467(0.642)
IDO1	−0.79(0.431)	−0.79(0.431)	−1.214(0.230)	2.804(0.006)	−1.513(0.132)	−0.313(0.755)
IL20RB	0.437(0.662)	0.437(0.662)	−1.909(0.061)	2.787(0.008)	−1.236(0.220)	−2.218(0.028)
PPP3CA	2.037(0.043)	2.037(0.043)	0.267(0.790)	−0.183(0.860)	−0.689(0.495)	−0.641(0.523)
PLAU	0.605(0.546)	0.605(0.546)	−1.422(0.158)	1.618(0.138)	−0.274(0.786)	0.807(0.424)
riskScore	0.039(0.969)	0.039(0.969)	−1.488(0.141)	2.007(0.047)	−1.943(0.054)	−1.533(0.127)

### Validation of the Prognosis-Associated Immune Signature for Pancreatic Ductal Adenocarcinoma in the Verification Cohort

**Figure 3:**
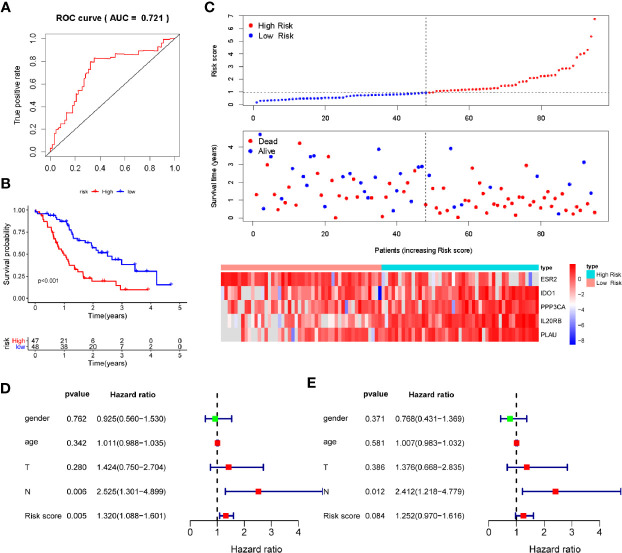
Validation of prognosis-associated immune signature (PAIS) for pancreatic ductal adenocarcinoma (PDAC) in the test cohort. **(A)** ROC curve analysis of the PAIS for overall survival (OS). **(B)** Kaplan-Meier survival curves of OS for patients with stages of PDAC based on PAIS. **(C)** Heatmap of 5-gene expression profiles, PAIS distributions, and live status of each patient in the high- and low-risk sets. **(D, E)** Univariate **(D)** and multivariate **(E)** Cox regression analyses of the associations between PAIS and clinical variables for the predictive value of OS.

To verify the discriminatory power of PAIS for PDAC, we applied the same formula to the test set consisting of 95 cases used in verification. All the prognosis-associated IRGs analyzed by Kaplan-Meier and Univariable Cox regression from ICGC database were shown in **Tables S5** and **S6**, respectively. Based on the cutoff values obtained from the training cohort, the 95 patients were separated to the low-set (n = 48) or high-risk set (n = 47) (**Figure 3C**). The PAIS for PDAC in the test cohort was identified as a robust prognostic model, with its AUC value at 1-year OS being found to be 0.721 (**Figure 3A**). Moreover, Kaplan-Meier survival analysis revealed that patients in High risk set PAIS also had significantly poorer OS than other patients (P < 0.001; **Figure 3B**). Next, we examined whether PAIS was an independent risk factor of OS in patients with PDAC in the test set. To this end, we performed univariate and multivariate Cox regression analyses using the data from these 95 patients. Our results revealed that both PAIS and lymph node metastasis not only served as predictor factors (PAIS: P = 0.005; lymph node metastasis: P = 0.006; **Figure 3D**; PAIS: P = 0.084; lymph node metastasis: P = 0.012; **Figure 3E**).

### Regulatory Network of Survival-Associated Immune-Related Genes

**Figure 4:**
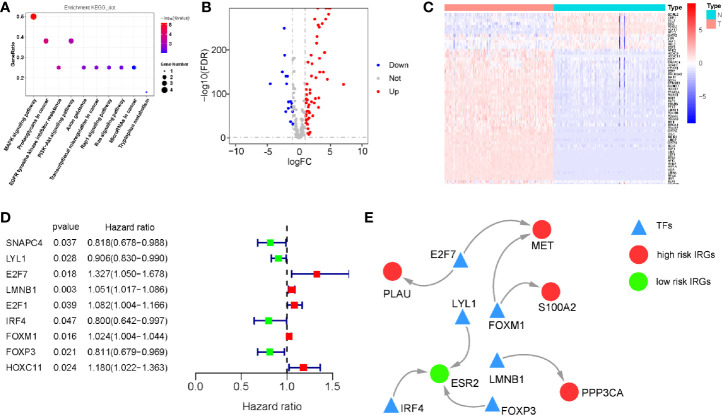
Regulatory network of survival-associated immune-related genes (IRGs). **(A)** Kyoto Encyclopedia of Genes and Genomes (KEGG) analysis (only show signaling pathway, P < 0.05). **(B)** Volcano plot demonstrating differentially expressed transcription factors (TFs) between pancreatic ductal adenocarcinoma (PDAC) and normal pancreatic tissues, Red dots represent genes with a higher level of expression in PDAC, whereas blue dots represent lower expression. **(C)** Heatmap showing the expression of TFs in each sample. **(D)** The hazard ratio and 95% confidence interval of survival-associated TFs using Cox proportional-hazards regression analysis. **(E)** Regulatory network constructed based on clinically relevant TFs and IRGs.

We investigated the regulatory mechanisms of survival-associated IRGs to explore their potential underlying molecular mechanisms. KEGG analysis showed that the MAPK and PI3K signaling pathways were the most enriched KEGG pathways, consistent with our results of differentially expressed IRGs (**Figure 4A**).

We then analyzed the expression of 318 TFs from the Cistrome Cancer database and found 73 TFs which were differentially expressed between PDAC and normal pancreatic tissue samples (**Figures 4B, C**). All the differentially expression TFs were shown in **Table S7**. Based on this, we identified the survival-associated differentially expressed TFs. The forest plot of hazard ratios and 95% CI (**Figure 4D**) in the Cox proportional hazards regression model showed that 9 TFs were correlated with the OS of patients with PDAC (P < 0.01). According to these survival-associated 9 TFs and 10 IRGs, we established a regulatory network(correlation score > 0.4 and P < 0.001). (**Figure 4E**).

### Association Between the Prognosis-Associated Immune Signature and Immune Response in Pancreatic Ductal Adenocarcinoma

**Figure 5:**
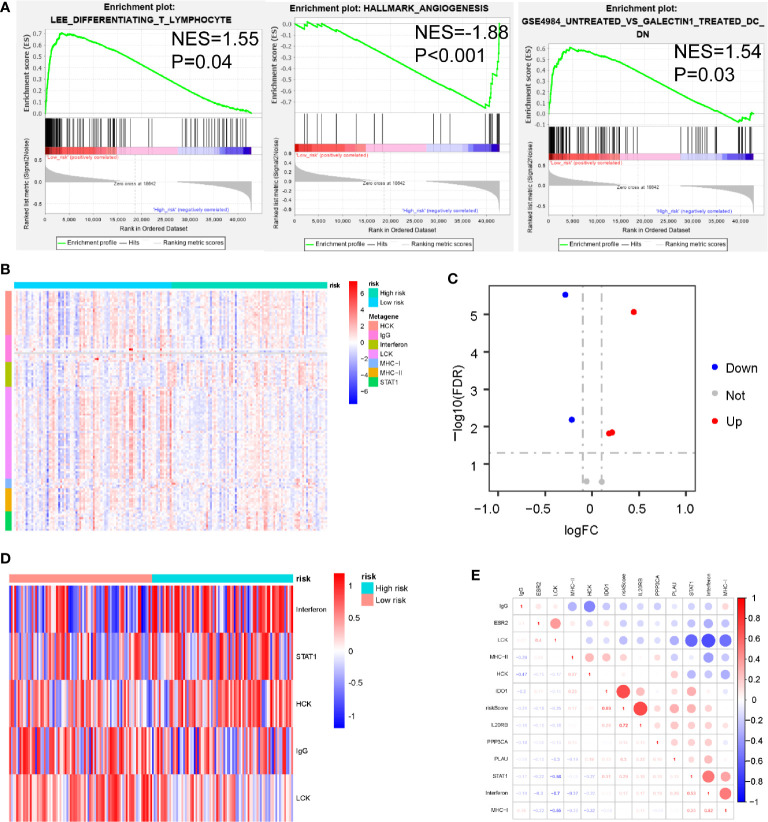
Associations between prognosis-associated immune signature (PAIS) and immune response in pancreatic ductal adenocarcinoma (PDAC). **(A)** Significant enrichment of immune pathways between the high-set and low-risk sets. NES: normalized enrichment score. **(B)** Heatmap of relationships between PAISs and seven clusters of inflammatory metagenes. **(C)** Volcano plot of four clusters of inflammatory metagenes differentially enriched in the high- and low-risk sets. **(D)** Heatmap of five clusters of inflammatory metagenes differentially enriched in the high- and low-risk sets. **(E)** Crosscorrelogram derived based on Pearson’s correlation coefficient values between PAISs and seven clusters of inflammatory metagenes.

To identify biological pathways related to risk score, we divided 163 PDAC samples obtained from the TCGA database into a high-set (n = 81) and low-risk set (n=82), based on the cut-off value (PAIS=0.933). We then performed GSEA to determine the distinct features of biological processes between these 2 sets. Our results indicated that patients with high-risk were strongly associated with a suppressive immune microenvironment, including higher angiogenesis (NES = 1.88, P < 0.001), naïve T-cells (NES = 1.55, P = 0.04), and DC cells with inhibitory function (NES = 1.54, P = 0.03) (**Figure 5A**).

For the better understanding of the association between PAIS and the immune response, we analyzed the expression of 7 previously described clusters of inflammatory metagenes (HCK, IgG, interferon, LCK, MHC-I, MHC-II, and STAT1) between the high- and low-risk sets (**Figure 5B**) (37). In addition, Gene set variation analysis (GSVA) was also performed to further explore the expression of these metagene clusters between the 2 sets. Our analysis revealed a powerful correlation between the PAIS and the hemopoietic cell kinase pathway, interferon response pathway, lymphocyte cell kinase pathway, IgG pathway, and STAT1 signal transduction pathway (**Figure 5C**) (38). The results for the expression level of 5 significantly differentially expressed metagene clusters in all samples between the 2 sets are presented in **Figure 5D**. To validate these findings and improve the interpretation of the observed associations, we used a cross correlogram to display the correlations among these variables. Our results prompted that the PAIS had positive associations with HCK, interferon, and STAT1, but negative associations with LCK and IgG (**Figure 5E**).

### Correlation Between the Prognosis-Associated Immune Signature and Immune Cell Infiltration or Immune Checkpoint Profiles in Pancreatic Ductal Adenocarcinoma

**Figure 6:**
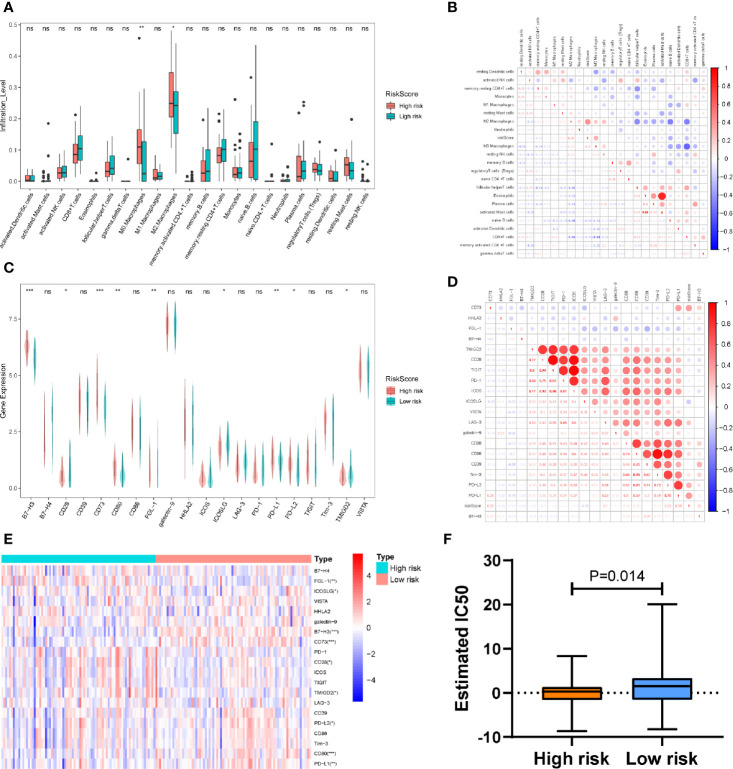
**(A)** Differences in the infiltration abundance of immune cells between the high-set and low-risk sets. **(B)** Cross correlogram derived based on Pearson’s correlation coefficient values between PAISs and 22 tumor-infiltrated immune cells. **(C)** Heatmap of immune checkpoint profiles in the high- and low-risk sets. **(D)** Differences in the expression of immune checkpoint molecules between the high-set and low-risk sets. **(E)** Cross correlogram derived based on Pearson’s correlation coefficient values between PAIS and the expression of immune checkpoint molecules. ns no significant, *P < 0.05, **P < 0.01, ***P < 0.001. **(F)** Boxplots evaluating the response to the paclitaxel chemotherapeutic between high-and low-risk patients.

Given that the immune response is closely related to the TIICs landscape, we chose a CIBERSORT algorithm to analyze the composition of TIICs between the high- and low-risk sets in patients with PDAC. Our results indicated that samples from high-risk patients were characterized by M0 macrophage and M2 macrophage enrichment (P < 0. 01; P<0.05) (**Figure 6A**). To further verify the association between the PAIS and the infiltration of TIICs, we used the cross correlogram to present the observed associations among these variables. Respectively, our results consistently indicated that the PAIS was positively correlated with the level of M2 macrophage infiltration (r = 0.43, P < 0.001), while negatively correlated with the level of CD8^+^ T-cells (r = −0.24, P = 0.042) (**Figure 6B**). The correlation between TIICS and clinical characters of PDAC patients is shown in **Table S8**.

Next, to gain new insights into the correlation between PAIS and immune checkpoint profiles, we included 16 immune checkpoint molecules in our analysis. We included the B7-CD28 family (CD28, CD80, CD86, ICOS, ICOSLG, PD-1, PD-L1, PD-L2, B7-H3, B7-H4, HHLA2, and TMIGD2) and several other hotspot immune checkpoint members (Tim-3, galectin-9, LAG-3 CD39, CD73, TIGIT, VISTA, and FGL-1) (**Figure 6C**) (39). Our results indicated that higher levels of CD80, PD-L1, B7-H3, and CD73 were expressed in the high-risk set (**Figures 6C, D**). We then used a cross correlogram to better understand the association between PAIS and the type of immune checkpoint molecules. Based on this analysis, we found that PAIS had a positive correlation with CD73 (r = 0.47, P < 0.001) and PD-L1 (r = 0.39, P < 0.001) (**Figure 6E**).

### Relationship Between the Prognosis-Associated Immune Signature and Drug Response in Pancreatic Ductal Adenocarcinoma

Given that chemotherapy is commonly used in the treatment of PDAC, we tried to evaluate the sensitivity of anticancer clinical drugs based on the evaluation of the expression level of tumor genes using the “pRRophetic” R package. After estimating the IC50 for each sample in the TCGA cohort, we selected paclitaxel and found that it had significant response sensitivities in high-risk cases rather than those in low-risk cases (P = 0.014; **Figure 6F**).

## Discussion

To date, a number of studies have revealed the molecular changings at different levels (such as DNA, RNA, protein, epigenetics, and TME) by using TCGA for the early screening and target therapy of PDAC in the future (3, 40–45). Immunotherapy has exhibited great potential in cancer treatment, but its effectiveness in patients with PDAC has not been satisfactory due to the highly immunosuppressive microenvironment of the tumor (46, 47). Pu et al. proposed the use of an immune and a stromal score based on the abundance of immune cells in order to characterize the TME of PDAC (48). They accordingly calculated the immune and stromal scores using an expression data (ESTIMATE) algorithm, and explored the relationships between the immune score and the PDAC subtype and mutant (TP53, KRAS, SMAD4, CDKN2A) cases. GSEA analysis also showed that PDAC samples have abnormal immune status. As the immune-related molecular mechanisms remain unclear in PDAC, our study described the immune status of PDAC using IRGs and TIICS.

We obtained differentially expressed IRGs from the TCGA and GTEx databases, and found ICGC-AU for testing. Using KEGG analysis, we first identified 832 differentially expressed IRGs enriched in pathways related to immune response and TME. We then identified a PAIS that was significantly related to the OS of patients with PDAC. We also used the transcriptome data analysis of a training cohort obtained from the TCGA database, which was also well validated in a test cohort from the ICGC-AU database.

Based on our analyses, 5 immune-related genes (ESR2, IDO1, IL20RB, PPP3CA, and PLAU) were identified and included in the prognostic model. We observed that IDO1, IL20RB, PPP3CA, and PLAU were negatively associated with favorable outcomes and were found to participate in tumor progression, whereas ESR2 showed the opposite effect. The IDO1 (indoleamine 2,3-dioxygenase 1) protein, one of the famous immune checkpoints, is the only rate-limiting enzyme that can catalyze the catabolism of tryptophan. Tryptophan is known to improve immune suppression of TME (49). In PDAC murine model, an IDO1 inhibitor was shown to improve the anti-tumor efficacy of GVAX (50). High expression of IDO1 has shown a positive correlation with immune tolerance and poor prognosis in patients with melanoma, glioma, and esophageal cancer (49, 51); this finding was, to some extent, consistent with our results. The IL20RB (interleukin-20 receptor subunit beta) protein, can bind to IL20 subfamily and activate JAK-STAT pathway.IL20RB also has shown a positive correlation trend with shorter survival in patients with several different types of tumors, including PDAC (52, 53). The PPP3CA (protein phosphatase 3 catalytic subunit alpha) protein, is a serine/threonine protein phosphatase responsible for the dephosphorylation of NFATc1, which is known to be indispensable for the activation of T-cells. Moreover, PPP3CA was also shown to serve as one of the predictor genes in melanoma (54). The PLAU (plasminogen activator urokinase) protein has been shown to mediate the Treg suppressor function *via* STAT5 and ERK signaling pathways (55, 56) and could provide certain help for future treatment in gastric cancer together with FOXM1. The ESR2 (estrogen receptor 2) protein, has been found to increase p53 signaling and apoptosis and reduce cell proliferation in colorectal cancer (57), but appeared to correlate with poor prognosis in resected PDAC (58). However, another report showed the opposite results (59). Our study was the first to reveal that low expression of IDO1, IL20RB, PPP3CA, and PLAU and high expression of ESR2 were correlated with an unfavorable prognosis in patients with PDAC. Stratification analysis and multivariate Cox analysis revealed that PAIS was an independent prognostic factor for OS in patients with PDAC.

To explore the functions and potential mechanisms of survival-associated IRGs, KEGG analysis and TF-regulated network has been performed. KEGG analysis showed that MAPK signaling pathway is the most significant one. Meanwhile, MAPK signaling pathway was one of the pathways which differentially expressed IRGs enriched in. The MAPK signaling pathway was found to regulate the expression level of PD-L1, the most promising immune checkpoint. Moreover, MAPK-targeted therapy might combination with T cell and TME, thus combined ICIs may show certain clinical benefits (60, 61). Therefore, PAIS might be able to predict the efficacy of MAPK inhibitors of patients with PDAC. On the other hand, after selecting the survival-associated TFs from DEGs, we finally selected LYL1, LMNB1, E2F7, FOXM1, IRF4, and FOXP3 that were observed to be prominently featured in this network. The correlation coefficient of IRF4-ESR2 was shown to be the highest (0.66). The IRF4 (interferon regulatory factor 4) protein is one of the key transcription factors of T-cell receptors (TCR) that might result in T-cell exhaustion (62). In particular, IRF4 has been reported to also promote the differentiation of naïve CD4^+^ T-cells (63), and has been shown to influence the T-cell-mediated immune responses. Although there have been no reports on the association of IRF4 and ESR2, infiltrating T-cells were reported to promote bladder cancer metastasis by regulation of the ESR2/c-MET or ESR2/IL-1/c-MET signaling pathways (64), whereas the secretion of interleukin-6 and tumor necrosis factor-alpha were correlated with Esr2 gene dose responses (65), suggesting that both IRF4 and ESR2 might influence the immune response. The association between IRF4 and ESR2 in PDAC was shown for the first time in our study.

To investigate the potential underlying mechanism of PAIS discriminating high- and low-risk patients, we executed GSEA to explore the distinct features of biological processes between these 2 sets. We found that high-risk patients were strongly associated with a suppressive immune microenvironment. This result indicated that immune heterogeneity between the 2 sets might be the key origin of the difference in overall prognosis. We therefore examined PAIS-associated immune variations between the 2 sets. Accordingly, we used analyses of 7 clusters of inflammatory metagenes, immune cell infiltration, and immune checkpoint profiles to provide additional insight into the immune landscapes associated with these 2 sets. We found that the PAIS had a positive relationship with the inflammatory response (HCK, STAT1, and interferon), but negative with T-cell and B-cell relative pathways (IgG and LCK). We further evaluated the expression level of many immunosuppressive checkpoint molecules, including PD-L1, galectin-9, B7-H3, PD-L2, CD80, and CD73. These results prompted that patients in the high-risk set were in an immunosuppressive state, so that immunotherapy may be less effective. Furthermore, the evaluation of TIICs, which are known to promote or regulate tumor progression and growth, is another important way to study the TME of PDAC. In the current study, CIBERSORT analysis revealed that monocytes and lymphocytes were higher in PDAC samples compared with granulocytes. We then explored the relationships between the risk score and TIICS infiltration and found the infiltration level of M2 macrophages was positively correlated with the risk score. M2 macrophages, which are also regarded as a tumor-associated macrophage (TAMs), have been reported as immune suppressive cells in TME (66). M2 macrophages have been suggested that infiltrated a higher level in high grade and invasive PDAC samples. M2 macrophages are known to express PD-L1, secrete immunosuppressive cytokines, chemokines, and enzymes, so that facilitate tumor angiogenesis and metastasis of PDAC (67–69). Meanwhile, the infiltration level of CD8^+^ T-cells was shown to be negatively correlated with the PAIS, indicating the presence of a relatively suppressed antitumor immune response state in high-risk patients. Therefore, we analyzed CD8 expression, CD8^+^ T cell infiltration and exhaustion related genes on the prognosis of PDAC patients in TCGA and ICGC database. The impact of CD8^+^ T cell infiltration and CD8 expression were not correlated with survival of PDAC. But according to recent study, the combination of PD-L1- and high CD8 expression identified a subtype with favorable survival (70), which is consistent with the expected our results (PAIS were higher in low CD8^+^ T cell infiltration and high PD-L1 group.) We also found that TGFB1 and TGFB2 were high risk factors in PDAC patients in ICGC database (**Table S5**). We hypothesized that the proportion of exhaustion T cells might be negatively associated with prognosis of PDAC patients. The present results indicated that low-risk sets may have the presence of a relatively active anti-tumor immune response state. Research on large-scale samples and comprehensive analysis of TIICS in PDAC is needed in the future.

Considering the clinical chemotherapy of patients with PDAC, we used the GDSC database and imputed that tumors from high-risk patients could be more sensitive to commonly used chemotherapy (e.g., paclitaxel). Therefore, high-risk patients could receive chemotherapy after curative surgery to obtain a longer survival benefit. However, it was unable to further validate the predictive power of PAIS in PDAC patients receiving neoadjuvant chemotherapy due to the limitations of the GEO database in this study. Nevertheless, prospective studies of clinical chemotherapy are necessary in the future.

In conclusion, we systematically analyzed the function and relevant signaling pathways of differentially expressed IRGs and their role in the prognosis of PDAC. IRGs could be useful in identifying new biomarkers, screen patients suitable for immunotherapy, and implement a novel PAIS model to predict the efficacy of targeted therapeutic approaches in the future. Further research *in vitro* or *in vivo* on IRGs might be helpful to better understand the microenvironment, immune evasion mechanisms, and novel immunotherapeutic targets in PDAC.

## Data Availability Statement

The original contributions presented in the study are included in the article/**Supplementary Material**. Further inquiries can be directed to the corresponding authors.

## Author Contributions

WW designed the study, performed the experiments, analyzed the data, and wrote the manuscript. LY, XG, JW, BD, and MZ performed the experiments and analyzed the data. CH and XT conceived and designed the study and wrote the manuscript. All authors contributed to the article and approved the submitted version.

## Funding

This study was supported by the Capital Health Research and Development of Special Funds (approval No.: 2020-1-1021), Beijing Municipal Natural Science Foundation (approval No. Z190022 and 7153161), Beijing Municipal Administration of Hospital’s Ascent Plan (grant no. DFL20181104), the National Natural Science Funding (grant no. 31770836), Interdisciplinary Medicine Seed Fund of Peking University and the Fundamental Research Funds for the Central Universities (approval No.: BMU2020MX015), and Science Foundation of Peking University Cancer Hospital 2020-13 and 2020-14.

## Conflict of Interest

The authors declare that the research was conducted in the absence of any commercial or financial relationships that could be construed as a potential conflict of interest.

## References

[1] Siegel RL Miller KD Jemal A. Cancer statistics, 2020. CA Cancer J Clin (2020) 70(1):7–30. 10.3322/caac.21590 31912902

[2] Li YJ Wu JY Wang JM Xiang DX. Emerging nanomedicine-based strategies for preventing metastasis of pancreatic cancer. J Control Release (2020) 320:105–11. 10.1016/j.jconrel.2020.01.041 31978441

[3] Kandimalla R Tomihara H Banwait JK Yamamura K Singh G Baba H. A 15-Gene Immune, Stromal, and Proliferation Gene Signature that Significantly Associates with Poor Survival in Patients with Pancreatic Ductal Adenocarcinoma. Clin Cancer Res (2020) 26(14):3641–8. 10.1158/1078-0432.CCR-19-4044 PMC7367725 32234757

[4] Kamisawa T Wood LD Itoi T Takaori K. Pancreatic cancer. Lancet (2016) 388(10039):73–85. 10.1016/S0140-6736(16)00141-0 26830752

[5] Topalian SL Hodi FS Brahmer JR Gettinger SN Smith DC McDermott DF. Safety, activity, and immune correlates of anti-PD-1 antibody in cancer. N Engl J Med (2012) 366(26):2443–54. 10.1056/NEJMoa1200690 PMC3544539 22658127

[6] Topalian SL Sznol M McDermott DF Kluger HM Carvajal RD Sharfman WH. Survival, durable tumor remission, and long-term safety in patients with advanced melanoma receiving nivolumab. J Clin Oncol (2014) 32(10):1020–30. 10.1200/JCO.2013.53.0105 PMC4811023 24590637

[7] Ansell SM Lesokhin AM Borrello I Halwani A Scott EC Gutierrez M. PD-1 blockade with nivolumab in relapsed or refractory Hodgkin’s lymphoma. N Engl J Med (2015) 372(4):311–9. 10.1056/NEJMoa1411087 PMC4348009 25482239

[8] Borghaei H Paz-Ares L Horn L Spigel DR Steins M Ready NE. Nivolumab versus Docetaxel in Advanced Nonsquamous Non-Small-Cell Lung Cancer. N Engl J Med (2015) 373(17):1627–39. 10.1056/NEJMoa1507643 PMC5705936 26412456

[9] Ribas A Wolchok JD. Cancer immunotherapy using checkpoint blockade. Science (2018) 359(6382):1350–5. 10.1126/science.aar4060 PMC7391259 29567705

[10] Ma J Sun D Wang J Han C Qian Y Chen G. Immune checkpoint inhibitors combined with chemotherapy for the treatment of advanced pancreatic cancer patients. Cancer Immunol Immunother (2020) 69(3):365–72. 10.1007/s00262-019-02452-3 PMC11027858 31897660

[11] Chung FF Mai CW Ng PY Leong CO. Cytochrome P450 2W1 (CYP2W1) in Colorectal Cancers. Curr Cancer Drug Targets (2016) 16(1):71–8. 10.2174/1568009616888151112095948 26563883

[12] Mai CW Chung FF Leong CO. Targeting Legumain As a Novel Therapeutic Strategy in Cancers. Curr Drug Targets (2017) 18(11):1259–68. 10.2174/1389450117666161216125344 27993111

[13] Mai CW Kang YB Pichika MR. Should a Toll-like receptor 4 (TLR-4) agonist or antagonist be designed to treat cancer? TLR-4: its expression and effects in the ten most common cancers. Onco Targets Ther (2013) 6:1573–87. 10.2147/OTT.S50838 PMC3821792 24235843

[14] Lutz E Yeo CJ Lillemoe KD Biedrzycki B Kobrin B Herman J. A lethally irradiated allogeneic granulocyte-macrophage colony stimulating factor-secreting tumor vaccine for pancreatic adenocarcinoma. A Phase II trial of safety, efficacy, and immune activation. Ann Surg (2011) 253(2):328–35. 10.1097/SLA.0b013e3181fd271c PMC3085934 21217520

[15] Thind K Padrnos LJ Ramanathan RK Borad MJ. Immunotherapy in pancreatic cancer treatment: a new frontier. Therap Adv Gastroenterol (2017) 10(1):168–94. 10.1177/1756283X16667909 PMC5330603 28286568

[16] Guo S Contratto M Miller G Leichman L Wu J. Immunotherapy in pancreatic cancer: Unleash its potential through novel combinations. World J Clin Oncol (2017) 8(3):230–40. 10.5306/wjco.v8.i3.230 PMC5465012 28638792

[17] Hurwitz HI Uppal N Wagner SA Bendell JC Beck JT Wade SM 3rd. Randomized, Double-Blind, Phase II Study of Ruxolitinib or Placebo in Combination With Capecitabine in Patients With Metastatic Pancreatic Cancer for Whom Therapy With Gemcitabine Has Failed. J Clin Oncol (2015) 33(34):4039–47. 10.1200/JCO.2015.61.4578 PMC5089161 26351344

[18] Ren B Cui M Yang G Wang H Feng M You L. Tumor microenvironment participates in metastasis of pancreatic cancer. Mol Cancer (2018) 17(1):108. 10.1186/s12943-018-0858-1 30060755 PMC6065152

[19] Chronopoulos A Robinson B Sarper M Cortes E Auernheimer V Lachowski D. ATRA mechanically reprograms pancreatic stellate cells to suppress matrix remodelling and inhibit cancer cell invasion. Nat Commun (2016) 7:12630. 10.1038/ncomms12630 27600527 PMC5023948

[20] Whiteside TL. The tumor microenvironment and its role in promoting tumor growth. Oncogene (2008) 27(45):5904–12. 10.1038/onc.2008.271 PMC3689267 18836471

[21] Dougan SK. The Pancreatic Cancer Microenvironment. Cancer J (2017) 23(6):321–5. 10.1097/PPO.0000000000000288 29189327

[22] Han J Chen M Wang Y Gong B Zhuang T Liang L. Identification of Biomarkers Based on Differentially Expressed Genes in Papillary Thyroid Carcinoma. Sci Rep (2018) 8(1):9912. 10.1038/s41598-018-28299-9 29967488 PMC6028435

[23] Yang H Zhang X Cai XY Wen DY Ye ZH Liang L. From big data to diagnosis and prognosis: gene expression signatures in liver hepatocellular carcinoma. PeerJ (2017) 5:e3089. 10.7717/peerj.3089 28316892 PMC5354077

[24] Li B Cui Y Diehn M Li R. Development and Validation of an Individualized Immune Prognostic Signature in Early-Stage Nonsquamous Non-Small Cell Lung Cancer. JAMA Oncol (2017) 3(11):1529–37. 10.1001/jamaoncol.2017.1609 PMC5710196 28687838

[25] Bhattacharya S Andorf S Gomes L Dunn P Schaefer H Pontius J. ImmPort: disseminating data to the public for the future of immunology. Immunol Res (2014) 58(2-3):234–9. 10.1007/s12026-014-8516-1 24791905

[26] Love MI Huber W Anders S. Moderated estimation of fold change and dispersion for RNA-seq data with DESeq2. Genome Biol (2014) 15(12):550. 10.1186/s13059-014-0550-8 25516281 PMC4302049

[27] Subramanian A Tamayo P Mootha VK Mukherjee S Ebert BL Gillette MA. Gene set enrichment analysis: a knowledge-based approach for interpreting genome-wide expression profiles. Proc Natl Acad Sci U S A (2005) 102(43):15545–50. 10.1073/pnas.0506580102 PMC1239896 16199517

[28] Liu J Lichtenberg T Hoadley KA Poisson LM Lazar AJ Cherniack AD. An Integrated TCGA Pan-Cancer Clinical Data Resource to Drive High-Quality Survival Outcome Analytics. Cell (2018) 173(2):400–16.e11. 10.1016/j.cell.2018.02.052 29625055 PMC6066282

[29] Karasinska JM Topham JT Kalloger SE Jang GH Denroche RE Culibrk L. Altered Gene Expression along the Glycolysis-Cholesterol Synthesis Axis Is Associated with Outcome in Pancreatic Cancer. Clin Cancer Res (2020) 26(1):135–46. 10.1158/1078-0432.CCR-19-1543 31481506

[30] Liu Y Wu L Ao H Zhao M Leng X Liu M. Prognostic implications of autophagy-associated gene signatures in non-small cell lung cancer. Aging (Albany NY) (2019) 11(23):11440–62. 10.18632/aging.102544 PMC6932887 31811814

[31] Long J Wang A Bai Y Lin J Yang X Wang D. Development and validation of a TP53-associated immune prognostic model for hepatocellular carcinoma. EBioMedicine (2019) 42:363–74. 10.1016/j.ebiom.2019.03.022 PMC6491941 30885723

[32] Mei S Meyer CA Zheng R Qin Q Wu Q Jiang P. Cistrome Cancer: A Web Resource for Integrative Gene Regulation Modeling in Cancer. Cancer Res (2017) 77(21):e19–22. 10.1158/0008-5472.CAN-17-0327 PMC5826647 29092931

[33] Newman AM Liu CL Green MR Gentles AJ Feng W Xu Y. Robust enumeration of cell subsets from tissue expression profiles. Nat Methods (2015) 12(5):453–7. 10.1038/nmeth.3337 PMC4739640 25822800

[34] Ge P Wang W Li L Zhang G Gao Z Tang Z. Profiles of immune cell infiltration and immune-related genes in the tumor microenvironment of colorectal cancer. BioMed Pharmacother (2019) 118:109228. 10.1016/j.biopha.2019.109228 31351430

[35] Yang X Shi Y Li M Lu T Xi J Lin Z. Identification and validation of an immune cell infiltrating score predicting survival in patients with lung adenocarcinoma. J Transl Med (2019) 17(1):217. 10.1186/s12967-019-1964-6 31286969 PMC6615164

[36] Geeleher P Cox NJ Huang RS. Clinical drug response can be predicted using baseline gene expression levels and in vitro drug sensitivity in cell lines. Genome Biol (2014) 15(3):R47. 10.1186/gb-2014-15-3-r47 24580837 PMC4054092

[37] Rody A Holtrich U Pusztai L Liedtke C Gaetje R Ruckhaeberle E. T-cell metagene predicts a favorable prognosis in estrogen receptor-negative and HER2-positive breast cancers. Breast Cancer Res (2009) 11(2):R15. 10.1186/bcr2234 19272155 PMC2688939

[38] Hanzelmann S Castelo R Guinney J. GSVA: gene set variation analysis for microarray and RNA-seq data. BMC Bioinformatics (2013) 14:7. 10.1186/1471-2105-14-7 23323831 PMC3618321

[39] Qin S Xu L Yi M Yu S Wu K Luo S. Novel immune checkpoint targets: moving beyond PD-1 and CTLA-4. Mol Cancer (2019) 18(1):155. 10.1186/s12943-019-1091-2 31690319 PMC6833286

[40] Yan Y Gao R Trinh TLP Grant MB. Immunodeficiency in Pancreatic Adenocarcinoma with Diabetes Revealed by Comparative Genomics. Clin Cancer Res (2017) 23(20):6363–73. 10.1158/1078-0432.CCR-17-0250 PMC6022738 28684632

[41] Cancer Genome Atlas Research Network. Electronic address aadhe, Cancer Genome Atlas Research N. Integrated Genomic Characterization of Pancreatic Ductal Adenocarcinoma. Cancer Cell (2017) 32(2):185–203.e13. 10.1158/0008-5472.CAN-16-0658 28810144 PMC5964983

[42] Ho J Li X Zhang L Liang Y Hu W Yau JCW. Translational genomics in pancreatic ductal adenocarcinoma: A review with re-analysis of TCGA dataset. Semin Cancer Biol (2019) 55:70–7. 10.1016/j.semcancer.2018.04.004 29705685

[43] Liu P Kong L Liang K Wu Y Jin H Song B. Identification of dissociation factors in pancreatic Cancer using a mass spectrometry-based proteomic approach. BMC Cancer (2020) 20(1):45. 10.1186/s12885-020-6522-3 31959150 PMC6971861

[44] Li T Liu Q Zhang R Liao Q Zhao Y. Identification of prognosis-related genes and construction of multi-regulatory networks in pancreatic cancer microenvironment by bioinformatics analysis. Cancer Cell Int (2020) 20:341. 10.1186/s12935-020-01426-1 32724299 PMC7382032

[45] Li T Liu Q Zhang R Liao Q Zhao Y. Correction to: Identification of prognosis-related genes and construction of multi-regulatory networks in pancreatic cancer microenvironment by bioinformatics analysis. Cancer Cell Int (2020) 20:397. 10.1186/s12935-020-01426-1 32724299 PMC7382032

[46] Feig C Gopinathan A Neesse A Chan DS Cook N Tuveson DA. The pancreas cancer microenvironment. Clin Cancer Res (2012) 18(16):4266–76. 10.1158/1078-0432.CCR-11-3114 PMC3442232 22896693

[47] Li M Li M Yang Y Liu Y Xie H Yu Q. Remodeling tumor immune microenvironment via targeted blockade of PI3K-gamma and CSF-1/CSF-1R pathways in tumor associated macrophages for pancreatic cancer therapy. J Control Release (2020) 321:23–35. 10.1016/j.jconrel.2020.02.011 32035193

[48] Pu N Chen Q Gao S Liu G Zhu Y Yin L. Genetic landscape of prognostic value in pancreatic ductal adenocarcinoma microenvironment. Ann Transl Med (2019) 7(22):645. 10.21037/atm.2019.10.91 31930046 PMC6944582

[49] Zhai L Ladomersky E Lenzen A Nguyen B Patel R Lauing KL. IDO1 in cancer: a Gemini of immune checkpoints. Cell Mol Immunol (2018) 15(5):447–57. 10.1038/cmi.2017.143 PMC6068130 29375124

[50] Blair AB Kleponis J Thomas DL 2nd Muth ST Murphy AG Kim V. IDO1 inhibition potentiates vaccine-induced immunity against pancreatic adenocarcinoma. J Clin Invest (2019) 129(4):1742–55. 10.1172/JCI124077 PMC6436883 30747725

[51] Kiyozumi Y Baba Y Okadome K Yagi T Ishimoto T Iwatsuki M. IDO1 Expression Is Associated With Immune Tolerance and Poor Prognosis in Patients With Surgically Resected Esophageal Cancer. Ann Surg (2019) 269(6):1101–8. 10.1097/SLA.0000000000002754 31082908

[52] Haider S Wang J Nagano A Desai A Arumugam P Dumartin L. A multi-gene signature predicts outcome in patients with pancreatic ductal adenocarcinoma. Genome Med (2014) 6(12):105. 10.1186/s13073-014-0105-3 25587357 PMC4293116

[53] Cui XF Cui XG Leng N. Overexpression of interleukin-20 receptor subunit beta (IL20RB) correlates with cell proliferation, invasion and migration enhancement and poor prognosis in papillary renal cell carcinoma. J Toxicol Pathol (2019) 32(4):245–51. 10.1293/tox.2019-0017 PMC6831501 31719751

[54] Tian M Yang J Han J He J Liao W. A novel immune checkpoint-related seven-gene signature for predicting prognosis and immunotherapy response in melanoma. Int Immunopharmacol (2020) 87:106821. 10.1016/j.intimp.2020.106821 32731180

[55] He F Chen H Probst-Kepper M Geffers R Eifes S Del Sol A. PLAU inferred from a correlation network is critical for suppressor function of regulatory T cells. Mol Syst Biol (2012) 8:624. 10.1038/msb.2012.56 23169000 PMC3531908

[56] Ai C Zhang J Lian S Ma J Gyorffy B Qian Z. FOXM1 functions collaboratively with PLAU to promote gastric cancer progression. J Cancer (2020) 11(4):788–94. 10.7150/jca.37323 PMC6959008 31949481

[57] Williams C DiLeo A Niv Y Gustafsson JA. Estrogen receptor beta as target for colorectal cancer prevention. Cancer Lett (2016) 372(1):48–56. 10.1016/j.canlet.2015.12.009 26708506 PMC4744541

[58] Seeliger H Pozios I Assmann G Zhao Y Muller MH Knosel T. Expression of estrogen receptor beta correlates with adverse prognosis in resected pancreatic adenocarcinoma. BMC Cancer (2018) 18(1):1049. 10.1186/s12885-018-4973-6 30373552 PMC6206939

[59] Pozios I Knosel T Zhao Y Assmann G Pozios I Muller MH. Expression of phosphorylated estrogen receptor beta is an independent negative prognostic factor for pancreatic ductal adenocarcinoma. J Cancer Res Clin Oncol (2018) 144(10):1887–97. 10.1007/s00432-018-2717-2 PMC11813511 30046904

[60] Zhang Y Velez-Delgado A Mathew E Li D Mendez FM Flannagan K. Myeloid cells are required for PD-1/PD-L1 checkpoint activation and the establishment of an immunosuppressive environment in pancreatic cancer. Gut (2017) 66(1):124–36. 10.1136/gutjnl-2016-312078 PMC5256390 27402485

[61] Shin MH Kim J Lim SA Kim J Lee KM. Current Insights into Combination Therapies with MAPK Inhibitors and Immune Checkpoint Blockade. Int J Mol Sci (2020) 21(7):2531. 10.3390/ijms21072531 PMC7177307 32260561

[62] Man K Gabriel SS Liao Y Gloury R Preston S Henstridge DC. Transcription Factor IRF4 Promotes CD8(+) T Cell Exhaustion and Limits the Development of Memory-like T Cells during Chronic Infection. Immunity (2017) 47(6):1129–41.e5. 10.1016/j.immuni.2017.11.021 29246443

[63] Huber M Lohoff M. IRF4 at the crossroads of effector T-cell fate decision. Eur J Immunol (2014) 44(7):1886–95. 10.1002/eji.201344279 24782159

[64] Tao L Qiu J Slavin S Ou Z Liu Z Ge J. Recruited T cells promote the bladder cancer metastasis via up-regulation of the estrogen receptor beta/IL-1/c-MET signals. Cancer Lett (2018) 430:215–23. 10.1016/j.canlet.2018.03.045 29684419

[65] Polanczyk M Yellayi S Zamora A Subramanian S Tovey M Vandenbark AA. Estrogen receptor-1 (Esr1) and -2 (Esr2) regulate the severity of clinical experimental allergic encephalomyelitis in male mice. Am J Pathol (2004) 164(6):1915–24. 10.1016/S0002-9440(10)63752-2 PMC1615766 15161628

[66] Mielgo A Schmid MC. Impact of tumour associated macrophages in pancreatic cancer. BMB Rep (2013) 46(3):131–8. 10.5483/BMBRep.2013.46.3.036 PMC4133870 23527856

[67] Hao NB Lu MH Fan YH Cao YL Zhang ZR Yang SM. Macrophages in tumor microenvironments and the progression of tumors. Clin Dev Immunol (2012) 2012:948098. 10.1155/2012/948098 22778768 PMC3385963

[68] Cui R Yue W Lattime EC Stein MN Xu Q Tan XL. Targeting tumor-associated macrophages to combat pancreatic cancer. Oncotarget (2016) 7(31):50735–54. 10.18632/oncotarget.9383 PMC5226617 27191744

[69] Clark CE Hingorani SR Mick R Combs C Tuveson DA Vonderheide RH. Dynamics of the immune reaction to pancreatic cancer from inception to invasion. Cancer Res (2007) 67(19):9518–27. 10.1158/0008-5472.CAN-07-0175 17909062

[70] Danilova L Ho WJ Zhu Q Vithayathil T De Jesus-Acosta A Azad NS. Programmed Cell Death Ligand-1 (PD-L1) and CD8 Expression Profiling Identify an Immunologic Subtype of Pancreatic Ductal Adenocarcinomas with Favorable Survival. Cancer Immunol Res (2019) 7(6):886–95. 10.1158/2326-6066.CIR-18-0822 PMC6548624 31043417

