# Supplementary material for: Identification of an Immune-Related Signature for Predicting Prognosis in Patients With Pancreatic Ductal Adenocarcinoma
Source: Front Oncol. 2021 Feb 24;10:618215. doi: 10.3389/fonc.2020.618215 (PMC7945593; doi:10.3389/fonc.2020.618215)
Supplement: Supplementary file 1 [file DataSheet_1.docx]

Supplementary Material

# Supplementary Figures and Tables

TableS1 Clinicopathological characteristics of the patient cohorts

| Variables | TCGA (n=163) | ICGC(n=95) |
| --- | --- | --- |
| **Age** |  |  |
| <65 | 73 (44.8%) | 40(42.1%) |
| ≥65 | 90 (55.2%) | 55(57.9%) |
| **gender** |  |  |
| male | 90(55.2%) | 50(52.6%) |
| female | 73(44.8%) | 45(47.4%) |
| **Grade** |  |  |
| G1 | 23(14.1%) | NA |
| G2 | 91(55.8%) | NA |
| G3 | 47(28.8%) | NA |
| G4 | 1(0.6%) | NA |
| Gx | 1(0.6%) | NA |
| **stage** |  |  |
| Ⅰ | 14(8.6%) | NA |
| Ⅱ | 140(85.9%) | NA |
| Ⅲ | 3(1.8%) | NA |
| Ⅳ | 4(2.5%) | NA |
| Unknown | 2(1.2%) |  |
| **T staging** |  |  |
| T1 | 5(3.1%) | 2(2.1%) |
| T2 | 18(11.0%) | 10(10.5%) |
| T3 | 136(83.4%) | 65(68.4%) |
| T4 | 3(1.8%) | 2(2.1%) |
| Tx | 1(0.6%) | 11(11.6%) |
| **N staging** |  |  |
| N0 | 43(26.4%) | 27(28.4%) |
| N1 | 119(73.0%) | 51(53.7%) |
| Nx | 1(0.6%) | 17(17.9%) |
| **M staging** |  |  |
| M0 | 76(44.6%) | 0 |
| M1 | 4(2.5%) | 5(5.3%) |
| Mx | 83 (50.9%) | 90(94.7) |

TableS2 Different expression IRGs between PDAC and normal pancreas

| **ID** | **log2FoldChange** | **P adj** |
| --- | --- | --- |
| TRBV5-6 | 2.75 | <0.001 |
| TRBV12-3 | 3.83 | <0.001 |
| SEMG1 | 4.59 | <0.001 |
| RETNLB | 2.86 | <0.001 |
| PTH | -3.42 | <0.001 |
| OPRK1 | 2.22 | <0.001 |
| INSL6 | -1.03 | <0.001 |
| INSL4 | 1.80 | <0.001 |
| IGLV4-3 | 2.11 | <0.001 |
| IGKV6D-21 | 4.62 | <0.001 |
| IGKV2D-24 | 4.22 | <0.001 |
| IGKV1-39 | 6.03 | <0.001 |
| IGHV4-4 | 5.46 | <0.001 |
| IGHV1-3 | 5.84 | <0.001 |
| IFNA1 | 2.38 | <0.001 |
| HTR3C | 3.97 | <0.001 |
| GIP | 3.61 | <0.001 |
| FGF21 | -3.03 | <0.001 |
| DEFA3 | -1.79 | <0.001 |
| CGB2 | 3.99 | <0.001 |
| CELA1 | -1.57 | <0.001 |
| SLC10A2 | 1.91 | <0.001 |
| CRABP1 | 2.00 | <0.001 |
| GALR3 | -4.22 | <0.001 |
| AGTR2 | -1.02 | <0.001 |
| TRBV11-2 | 2.32 | <0.001 |
| DCD | -5.99 | <0.001 |
| NPPB | -2.49 | <0.001 |
| IGLV5-52 | -1.08 | <0.001 |
| PRL | -6.10 | <0.001 |
| GH1 | -6.77 | <0.001 |
| IL12B | 3.71 | <0.001 |
| CR2 | 7.45 | <0.001 |
| LPA | -2.91 | <0.001 |
| ACTA1 | -5.19 | <0.001 |
| FGF3 | 3.07 | <0.001 |
| CALCR | 2.58 | <0.001 |
| PRLHR | -1.65 | <0.001 |
| IL17C | 2.39 | <0.001 |
| FCN2 | -2.56 | <0.001 |
| NOS1 | -2.32 | <0.001 |
| EPO | -4.55 | <0.001 |
| NTS | 4.54 | <0.001 |
| IL22RA2 | 5.58 | <0.001 |
| CCL25 | 3.51 | <0.001 |
| FLG | -2.22 | <0.001 |
| IFNE | 5.07 | <0.001 |
| CLC | 1.74 | <0.001 |
| ANGPTL5 | 1.14 | <0.001 |
| RXFP3 | 1.04 | <0.001 |
| IGKV1D-8 | 4.91 | <0.001 |
| IGKV1D-17 | 3.83 | <0.001 |
| IFNG | 2.75 | <0.001 |
| HTR3E | 2.95 | <0.001 |
| CMA1 | 1.37 | <0.001 |
| TRGC1 | 1.92 | <0.001 |
| NPPC | 2.41 | <0.001 |
| TDGF1 | 3.05 | <0.001 |
| TRBV4-2 | 3.08 | <0.001 |
| S100G | 1.56 | <0.001 |
| MCHR1 | 3.41 | <0.001 |
| IL5RA | 1.64 | <0.001 |
| DEFA5 | 1.44 | <0.001 |
| GDF10 | -4.69 | <0.001 |
| TRBV2 | 3.54 | <0.001 |
| IGKV3-7 | 4.41 | <0.001 |
| RORB | 1.24 | <0.001 |
| OBP2A | 3.80 | <0.001 |
| ARG1 | -2.17 | <0.001 |
| IL12RB2 | -3.17 | <0.001 |
| TRBV9 | 3.14 | <0.001 |
| TRAV13-2 | 3.03 | <0.001 |
| ULBP1 | 2.03 | <0.001 |
| GDF5 | 3.26 | <0.001 |
| FGF17 | -3.37 | <0.001 |
| TRBV24-1 | 1.75 | <0.001 |
| TRBV10-3 | 3.17 | <0.001 |
| TRAV8-6 | 3.26 | <0.001 |
| TRAV8-2 | 3.23 | <0.001 |
| CHP2 | 4.12 | <0.001 |
| CCR9 | 2.69 | <0.001 |
| TACR1 | 1.74 | <0.001 |
| TNFRSF13B | 4.47 | <0.001 |
| CD19 | 5.40 | <0.001 |
| TRBV19 | 3.85 | <0.001 |
| TRAV2 | 3.15 | <0.001 |
| TRAV13-1 | 3.51 | <0.001 |
| KIR2DL4 | 2.42 | <0.001 |
| NOS2 | 2.26 | <0.001 |
| FASLG | 2.20 | <0.001 |
| TRAV3 | 3.12 | <0.001 |
| WFIKKN1 | -3.09 | <0.001 |
| LTA | 3.16 | <0.001 |
| IL19 | 3.61 | <0.001 |
| FABP2 | 4.61 | <0.001 |
| CRHR1 | 2.16 | <0.001 |
| CCR3 | 3.55 | <0.001 |
| S100Z | 1.20 | <0.001 |
| ESRRB | -1.44 | <0.001 |
| GDF7 | -1.24 | <0.001 |
| SHC3 | -2.89 | <0.001 |
| IL17B | 2.79 | <0.001 |
| SH2D1B | 2.95 | <0.001 |
| CCL8 | 2.84 | <0.001 |
| PRLR | -2.35 | <0.001 |
| ADCYAP1R1 | -1.78 | <0.001 |
| CGA | -3.14 | <0.001 |
| GPHA2 | -3.52 | <0.001 |
| FGF5 | 4.01 | <0.001 |
| TRBV18 | 3.69 | <0.001 |
| IGHV3-20 | 4.64 | <0.001 |
| GLP1R | -2.67 | <0.001 |
| LCN1 | 2.49 | <0.001 |
| TRAV12-3 | 3.33 | <0.001 |
| PTH2R | -1.53 | <0.001 |
| CRHR2 | -4.43 | <0.001 |
| XCL1 | 1.88 | <0.001 |
| ESR2 | -2.24 | <0.001 |
| TRBV27 | 3.22 | <0.001 |
| TRAV8-4 | 3.16 | <0.001 |
| XCR1 | 3.57 | <0.001 |
| OBP2B | 2.16 | <0.001 |
| IGLV10-54 | 4.20 | <0.001 |
| TNFRSF13C | 4.64 | <0.001 |
| FGF10 | -1.56 | <0.001 |
| VIPR2 | -4.02 | <0.001 |
| NCR3 | 4.16 | <0.001 |
| IFNL1 | 2.39 | <0.001 |
| CD28 | 3.89 | <0.001 |
| PYY | 1.38 | <0.001 |
| GDF9 | -2.11 | <0.001 |
| NPPA | -4.77 | <0.001 |
| TNFSF18 | 3.46 | <0.001 |
| ICOS | 3.95 | <0.001 |
| IL1A | 4.75 | <0.001 |
| CD244 | 1.96 | <0.001 |
| IGHV1-58 | 5.95 | <0.001 |
| IGKV1-12 | 5.83 | <0.001 |
| TRAV17 | 3.51 | <0.001 |
| SEMA3D | 2.64 | <0.001 |
| TRBV6-1 | 2.32 | <0.001 |
| TRAV26-1 | 3.44 | <0.001 |
| CD40LG | 3.40 | <0.001 |
| TLR7 | 2.88 | <0.001 |
| MASP2 | -2.91 | <0.001 |
| XCL2 | 2.60 | <0.001 |
| CCL7 | 4.05 | <0.001 |
| IL10 | 1.27 | <0.001 |
| NRG2 | -2.56 | <0.001 |
| CYSLTR2 | 3.19 | <0.001 |
| TRGV5 | 1.56 | <0.001 |
| SH2D1A | 3.99 | <0.001 |
| AMHR2 | -3.79 | <0.001 |
| IL24 | 2.84 | <0.001 |
| TAC1 | 1.73 | <0.001 |
| CALCB | -2.58 | <0.001 |
| KLRC3 | 1.19 | <0.001 |
| PGR | 1.28 | <0.001 |
| CAMP | 2.74 | <0.001 |
| TRBV5-1 | 3.78 | <0.001 |
| PMCH | 4.42 | <0.001 |
| RAET1G | 2.15 | <0.001 |
| TNFRSF8 | 2.66 | <0.001 |
| COLEC10 | 3.04 | <0.001 |
| CSPG5 | 2.04 | <0.001 |
| IL13RA2 | 2.18 | <0.001 |
| PTGER1 | 1.18 | <0.001 |
| GDNF | 3.19 | <0.001 |
| THPO | -1.77 | <0.001 |
| CX3CR1 | 1.21 | <0.001 |
| FGF12 | -1.70 | <0.001 |
| TRBV3-1 | 3.36 | <0.001 |
| CGB8 | 9.60 | <0.001 |
| IL12A | -1.06 | <0.001 |
| FGF14 | 1.65 | <0.001 |
| TRAV12-2 | 3.69 | <0.001 |
| AMH | -1.47 | <0.001 |
| BDNF | 2.18 | <0.001 |
| TPO | -1.68 | <0.001 |
| EGF | -4.72 | <0.001 |
| CGB7 | 3.61 | <0.001 |
| CGB5 | 11.03 | <0.001 |
| RAET1L | 4.69 | <0.001 |
| IL1RL2 | 1.33 | <0.001 |
| TG | -2.47 | <0.001 |
| LCN6 | -2.44 | <0.001 |
| IGKV3D-11 | 5.41 | <0.001 |
| CD209 | 1.56 | <0.001 |
| NRG1 | 1.31 | <0.001 |
| TRDC | 2.93 | <0.001 |
| GCGR | -1.40 | <0.001 |
| GPR17 | -1.70 | <0.001 |
| CABP1 | -1.22 | <0.001 |
| TNFRSF17 | 3.91 | <0.001 |
| UTS2 | 4.58 | <0.001 |
| PDCD1 | 2.52 | <0.001 |
| TRBV7-3 | 1.91 | <0.001 |
| TRAV9-2 | 3.74 | <0.001 |
| APOBEC3H | 1.81 | <0.001 |
| TRBV7-9 | 3.32 | <0.001 |
| RAET1E | 1.90 | <0.001 |
| TNFSF15 | 3.41 | <0.001 |
| FGF20 | 2.94 | <0.001 |
| LEAP2 | -1.83 | <0.001 |
| TRBV6-6 | 1.54 | <0.001 |
| CCR4 | 4.89 | <0.001 |
| TFR2 | 1.07 | <0.001 |
| UCN2 | 5.12 | <0.001 |
| PNOC | 4.81 | <0.001 |
| RBP2 | 1.41 | <0.001 |
| TNFSF14 | -1.35 | <0.001 |
| PF4 | 4.39 | <0.001 |
| CCR8 | 4.93 | <0.001 |
| CD1B | 3.47 | <0.001 |
| AZU1 | 1.10 | <0.001 |
| PRKCB | 2.79 | <0.001 |
| IGF1 | 1.21 | <0.001 |
| TRBV12-4 | 3.22 | <0.001 |
| RNASE7 | 1.52 | <0.001 |
| PGLYRP3 | 2.08 | <0.001 |
| LGR6 | 4.79 | <0.001 |
| PENK | 3.25 | <0.001 |
| TRAV19 | 3.20 | <0.001 |
| SHC4 | 1.98 | <0.001 |
| PAK3 | -3.22 | <0.001 |
| GAL | 1.22 | <0.001 |
| KLKB1 | -2.25 | <0.001 |
| LTB4R2 | -1.97 | <0.001 |
| IGLC6 | 3.76 | <0.001 |
| IL9R | 1.80 | <0.001 |
| CXCL13 | 6.63 | <0.001 |
| GRAP2 | 1.60 | <0.001 |
| DEFA1 | -1.61 | <0.001 |
| LHB | -2.81 | <0.001 |
| TRAV21 | 3.16 | <0.001 |
| IGLV5-37 | 4.52 | <0.001 |
| IGKV3D-15 | 6.17 | <0.001 |
| TNFRSF9 | 3.96 | <0.001 |
| FLT3 | -1.13 | <0.001 |
| IGHD | 4.84 | <0.001 |
| BMP6 | 1.70 | <0.001 |
| CCR10 | -1.31 | <0.001 |
| ESRRG | -1.54 | <0.001 |
| PTX3 | -1.24 | <0.001 |
| NTF4 | 5.31 | <0.001 |
| NDP | 5.27 | <0.001 |
| NR6A1 | -1.47 | <0.001 |
| IGHV3-53 | 5.04 | <0.001 |
| GAST | 2.49 | <0.001 |
| FGF18 | 2.29 | <0.001 |
| CETP | 1.48 | <0.001 |
| IGHV4-28 | 3.73 | <0.001 |
| LCN10 | -2.32 | <0.001 |
| IGKV2D-30 | 4.68 | <0.001 |
| CTLA4 | 3.89 | <0.001 |
| RASGRP1 | 1.64 | <0.001 |
| CYSLTR1 | 1.52 | <0.001 |
| SEMA6D | -2.97 | <0.001 |
| POMC | -3.64 | <0.001 |
| IGKV1D-13 | 5.13 | <0.001 |
| MASP1 | 1.94 | <0.001 |
| LGR5 | 5.35 | <0.001 |
| IGHV3-43 | 5.54 | <0.001 |
| CD3G | 3.38 | <0.001 |
| PGC | 2.17 | <0.001 |
| ULBP3 | 2.22 | <0.001 |
| BMP3 | 3.32 | <0.001 |
| GREM2 | 3.20 | <0.001 |
| ITK | 2.57 | <0.001 |
| S100A12 | -1.15 | <0.001 |
| IL6 | 1.73 | <0.001 |
| C5 | -3.25 | <0.001 |
| IGHE | 5.81 | <0.001 |
| TRBV28 | 3.82 | <0.001 |
| SEMA3A | 3.82 | <0.001 |
| APLN | 2.37 | <0.001 |
| TMPRSS6 | 1.81 | <0.001 |
| IGHV2-70 | 5.09 | <0.001 |
| IGKV3D-20 | 5.62 | <0.001 |
| ANGPTL7 | 2.29 | <0.001 |
| TMSB15A | 1.05 | <0.001 |
| CD8B | 2.20 | <0.001 |
| IGHV3-64 | 4.45 | <0.001 |
| IGKV2D-40 | 5.42 | <0.001 |
| IGKV2-40 | 3.34 | <0.001 |
| AGRP | 3.64 | <0.001 |
| TRGC2 | 1.94 | <0.001 |
| SERPIND1 | 1.91 | <0.001 |
| CXCR6 | 2.11 | <0.001 |
| IGHV1-69 | 6.45 | <0.001 |
| SPINK5 | 2.53 | <0.001 |
| PAEP | 7.54 | <0.001 |
| INSL3 | 1.77 | <0.001 |
| DTL | 4.02 | <0.001 |
| TNF | 2.05 | <0.001 |
| NOD2 | 1.75 | <0.001 |
| IGLV3-27 | 5.43 | <0.001 |
| NR4A3 | 1.24 | <0.001 |
| NRG4 | -3.11 | <0.001 |
| TLR8 | 2.51 | <0.001 |
| IL7 | 1.60 | <0.001 |
| CCR5 | 2.33 | <0.001 |
| EBI3 | 1.61 | <0.001 |
| C8G | 1.18 | <0.001 |
| CD79A | 5.78 | <0.001 |
| IL21R | 4.01 | <0.001 |
| PIK3CG | 3.07 | <0.001 |
| IGLV3-9 | 5.52 | <0.001 |
| ACVR2B | -1.24 | <0.001 |
| IGLV2-18 | 4.17 | <0.001 |
| IGKV1D-16 | 4.21 | <0.001 |
| CCR7 | 3.88 | <0.001 |
| CXCR1 | 1.55 | <0.001 |
| TNFSF8 | 2.87 | <0.001 |
| FGF2 | 2.14 | <0.001 |
| OGN | 1.97 | <0.001 |
| ZAP70 | 1.51 | <0.001 |
| IL33 | 1.50 | <0.001 |
| IGHV3-13 | 5.48 | <0.001 |
| IL17RD | 1.43 | <0.001 |
| LCN12 | -1.20 | <0.001 |
| TRBV20-1 | 4.13 | <0.001 |
| IL2RB | 3.12 | <0.001 |
| CD22 | 3.51 | <0.001 |
| IGKV1D-33 | 6.15 | <0.001 |
| LIFR | -1.89 | <0.001 |
| CD70 | 5.34 | <0.001 |
| CTSG | 1.52 | <0.001 |
| CXCR2 | 1.98 | <0.001 |
| INHA | -2.17 | <0.001 |
| CCR6 | 6.53 | <0.001 |
| FGF11 | 1.68 | <0.001 |
| NTF3 | 1.64 | <0.001 |
| IGHV3-66 | 4.89 | <0.001 |
| VAV3 | -1.09 | <0.001 |
| PGLYRP4 | 2.19 | <0.001 |
| SEMA3E | 2.26 | <0.001 |
| HGF | 1.22 | <0.001 |
| IGKV5-2 | 3.82 | <0.001 |
| ROBO2 | 1.39 | <0.001 |
| IL11 | 4.08 | <0.001 |
| IGKV6-21 | 5.66 | <0.001 |
| TNFRSF18 | 3.64 | <0.001 |
| IL1B | 1.90 | <0.001 |
| PI15 | 3.09 | <0.001 |
| IGKV2-30 | 3.96 | <0.001 |
| TNFSF9 | 5.37 | <0.001 |
| CRLF2 | 2.48 | <0.001 |
| HAMP | -3.45 | <0.001 |
| CSF2 | 6.50 | <0.001 |
| GZMB | 2.91 | <0.001 |
| PRF1 | 2.10 | <0.001 |
| BPI | 3.19 | <0.001 |
| CD8A | 1.91 | <0.001 |
| IGLV9-49 | 4.86 | <0.001 |
| HLA-DOB | 3.35 | <0.001 |
| FGF13 | 1.31 | <0.001 |
| TNFRSF11A | 2.18 | <0.001 |
| IL12RB1 | 2.85 | <0.001 |
| FABP6 | 5.34 | <0.001 |
| IGHV1-46 | 5.18 | <0.001 |
| CD79B | 3.71 | <0.001 |
| CD1D | 1.34 | <0.001 |
| S100A5 | 4.78 | <0.001 |
| PLA2G2A | 2.44 | <0.001 |
| CXCR3 | 3.68 | <0.001 |
| IGLV1-36 | 5.19 | <0.001 |
| CMTM1 | 2.20 | <0.001 |
| NR0B2 | -1.50 | <0.001 |
| UCN3 | 1.21 | <0.001 |
| CD247 | 2.97 | <0.001 |
| IGHV3-48 | 4.44 | <0.001 |
| TSLP | 2.60 | <0.001 |
| STC2 | -2.19 | <0.001 |
| CD1E | 3.76 | <0.001 |
| IGLV3-10 | 4.43 | <0.001 |
| CCL24 | 6.35 | <0.001 |
| ITGAL | 2.43 | <0.001 |
| CXCL9 | 5.11 | <0.001 |
| GNLY | 2.09 | <0.001 |
| RNASE2 | 1.16 | <0.001 |
| FGF1 | 3.04 | <0.001 |
| IGHV6-1 | 5.15 | <0.001 |
| NRG3 | 5.27 | <0.001 |
| CXCL10 | 4.18 | <0.001 |
| IGLC7 | 5.17 | <0.001 |
| IGHV4-31 | 6.71 | <0.001 |
| TNFRSF4 | 1.80 | <0.001 |
| IGKV1-8 | 5.24 | <0.001 |
| CCR1 | 2.01 | <0.001 |
| ULBP2 | 3.21 | <0.001 |
| MME | 3.00 | <0.001 |
| SLIT2 | 1.44 | <0.001 |
| PTN | 1.16 | <0.001 |
| CCL15 | 5.02 | <0.001 |
| PTGER4 | -1.83 | <0.001 |
| CCL22 | 5.76 | <0.001 |
| IGHV2-26 | 5.96 | <0.001 |
| OXTR | 3.52 | <0.001 |
| APOBEC3F | 1.20 | <0.001 |
| IL23A | 2.17 | <0.001 |
| IDO1 | 5.11 | <0.001 |
| RORA | -1.31 | <0.001 |
| HSPA6 | 2.21 | <0.001 |
| NFATC2 | 1.40 | <0.001 |
| KLRC2 | 2.01 | <0.001 |
| IGKV2-24 | 4.33 | <0.001 |
| NRTN | -2.90 | <0.001 |
| NR5A2 | -2.54 | <0.001 |
| ESM1 | 3.99 | <0.001 |
| PDIA2 | -4.72 | <0.001 |
| SLIT3 | 1.45 | <0.001 |
| CMTM2 | 1.45 | <0.001 |
| NR1I2 | 4.22 | <0.001 |
| IGLV7-43 | 4.37 | <0.001 |
| APLNR | 3.03 | <0.001 |
| IGHV4-61 | 5.75 | <0.001 |
| IGHV4-34 | 5.46 | <0.001 |
| OSM | 2.94 | <0.001 |
| CCK | 4.49 | <0.001 |
| LTF | 1.70 | <0.001 |
| CD1A | 6.66 | <0.001 |
| TLR4 | 1.75 | <0.001 |
| FCGR3B | 2.17 | <0.001 |
| IGHV3-73 | 4.90 | <0.001 |
| CCL4 | 2.05 | <0.001 |
| APOBEC3G | 2.28 | <0.001 |
| DMBT1 | 5.47 | <0.001 |
| CALCRL | 1.82 | <0.001 |
| IGHV3-72 | 4.62 | <0.001 |
| CCL26 | 3.78 | <0.001 |
| IGLV5-45 | 4.18 | <0.001 |
| TNFSF13B | 2.05 | <0.001 |
| PTH1R | -1.28 | <0.001 |
| TNFSF4 | 2.57 | <0.001 |
| IGKV1-27 | 5.32 | <0.001 |
| IL27RA | 1.54 | <0.001 |
| TNFRSF19 | 1.16 | <0.001 |
| CSF2RB | 2.32 | <0.001 |
| MICB | 2.24 | <0.001 |
| IGLV4-60 | 5.01 | <0.001 |
| NOX1 | 6.39 | <0.001 |
| IGLV6-57 | 5.15 | <0.001 |
| CCL3 | 2.66 | <0.001 |
| IGLV4-69 | 4.55 | <0.001 |
| HLA-G | 2.72 | <0.001 |
| TNFRSF10C | 1.60 | <0.001 |
| KITLG | 1.58 | <0.001 |
| FLT3LG | 1.24 | <0.001 |
| AQP9 | 2.58 | <0.001 |
| PCSK2 | 1.08 | <0.001 |
| TMSB15B | 1.03 | <0.001 |
| CD1C | 4.00 | <0.001 |
| VEGFC | 2.19 | <0.001 |
| PTHLH | 4.14 | <0.001 |
| IL2RA | 4.97 | <0.001 |
| IL16 | 1.87 | <0.001 |
| BTK | 2.41 | <0.001 |
| ERAP2 | 2.52 | <0.001 |
| HLA-DQA2 | 4.43 | <0.001 |
| EPOR | -2.12 | <0.001 |
| BIRC5 | 3.71 | <0.001 |
| IL1RAP | 2.40 | <0.001 |
| RNASEL | 1.25 | <0.001 |
| IGHV2-5 | 4.97 | <0.001 |
| RSAD2 | 2.48 | <0.001 |
| IGHV3-49 | 4.64 | <0.001 |
| TGFBR1 | 1.53 | <0.001 |
| IGKV1-6 | 4.82 | <0.001 |
| BMP7 | 3.15 | <0.001 |
| CD86 | 3.24 | <0.001 |
| COLEC12 | 2.14 | <0.001 |
| SCT | 3.77 | <0.001 |
| IL34 | 1.40 | <0.001 |
| RARB | 2.04 | <0.001 |
| PIK3R5 | 1.28 | <0.001 |
| C3AR1 | 1.94 | <0.001 |
| CIITA | 1.42 | <0.001 |
| FGF19 | 7.86 | <0.001 |
| IGHV3-74 | 4.53 | <0.001 |
| IGLV7-46 | 4.42 | <0.001 |
| CD48 | 3.74 | <0.001 |
| CXCR5 | 2.10 | <0.001 |
| SCG2 | 1.30 | <0.001 |
| CD3E | 3.23 | <0.001 |
| ROR2 | 2.19 | <0.001 |
| BMP2 | 1.17 | <0.001 |
| CCL4L2 | 3.19 | <0.001 |
| SOCS1 | 1.40 | <0.001 |
| PTGER2 | 3.12 | <0.001 |
| PTPRC | 2.75 | <0.001 |
| TIE1 | 1.34 | <0.001 |
| SEMA6B | 1.12 | <0.001 |
| IL7R | 2.87 | <0.001 |
| IL10RA | 1.74 | <0.001 |
| NFATC1 | 1.22 | <0.001 |
| CCRL2 | 2.46 | <0.001 |
| CCL23 | 1.55 | <0.001 |
| RORC | -2.07 | <0.001 |
| FPR2 | 2.50 | <0.001 |
| REG3G | -3.39 | <0.001 |
| IKBKE | 1.39 | <0.001 |
| CCL13 | 3.66 | <0.001 |
| NOX4 | 4.40 | <0.001 |
| DCK | 1.36 | <0.001 |
| CCL19 | 4.44 | <0.001 |
| FAS | 1.44 | <0.001 |
| FGFR3 | -2.14 | <0.001 |
| SEMA3F | 1.31 | <0.001 |
| INPP5D | 2.16 | <0.001 |
| IGHV3-11 | 5.30 | <0.001 |
| DLL4 | 1.38 | <0.001 |
| TCF4 | 1.62 | <0.001 |
| CXCL2 | -2.45 | <0.001 |
| CD72 | 1.27 | <0.001 |
| IAPP | -1.34 | <0.001 |
| CXCL3 | 2.32 | <0.001 |
| PLXNA2 | -1.95 | <0.001 |
| IGKV2D-29 | 4.91 | <0.001 |
| IGLV2-8 | 4.08 | <0.001 |
| HLA-DOA | 3.15 | <0.001 |
| PLXNC1 | 2.43 | <0.001 |
| PDGFB | 1.49 | <0.001 |
| PGF | 1.62 | <0.001 |
| ROBO1 | 2.57 | <0.001 |
| PTGS2 | 4.85 | <0.001 |
| VAV1 | 3.00 | <0.001 |
| PROC | 3.73 | <0.001 |
| F2RL1 | 1.57 | <0.001 |
| CYBB | 2.37 | <0.001 |
| IGHV4-39 | 5.70 | <0.001 |
| GNAI1 | 1.49 | <0.001 |
| IGLV8-61 | 4.45 | <0.001 |
| SDS | 3.77 | <0.001 |
| CCL21 | 3.34 | <0.001 |
| HCK | 2.37 | <0.001 |
| STC1 | 1.83 | <0.001 |
| CXCL12 | -1.45 | <0.001 |
| IGKV1-16 | 5.27 | <0.001 |
| MR1 | 1.97 | <0.001 |
| CMTM8 | -1.44 | <0.001 |
| IGHV3-21 | 5.74 | <0.001 |
| FCGR2B | 2.80 | <0.001 |
| IGHV3-15 | 4.90 | <0.001 |
| IGHV4-59 | 5.52 | <0.001 |
| BMPR2 | 1.62 | <0.001 |
| EDNRA | 3.75 | <0.001 |
| IGKV1-9 | 5.29 | <0.001 |
| RFX5 | 1.12 | <0.001 |
| IGKV2D-28 | 7.09 | <0.001 |
| IGLV1-44 | 5.03 | <0.001 |
| TRAC | 3.75 | <0.001 |
| BCL10 | 1.89 | <0.001 |
| IGHV1-18 | 6.05 | <0.001 |
| SEMA7A | 5.10 | <0.001 |
| NPR3 | 3.61 | <0.001 |
| ALB | -4.76 | <0.001 |
| IL20RB | 3.98 | <0.001 |
| CHIT1 | 5.25 | <0.001 |
| APOBEC3C | 2.17 | <0.001 |
| TNFRSF1B | 2.04 | <0.001 |
| IGHV3-7 | 4.41 | <0.001 |
| LGR4 | -1.55 | <0.001 |
| EREG | 4.82 | <0.001 |
| RETN | 3.44 | <0.001 |
| LCP2 | 1.99 | <0.001 |
| PI3 | 6.20 | <0.001 |
| IGLV3-21 | 6.22 | <0.001 |
| VDR | 2.96 | <0.001 |
| SECTM1 | 1.74 | <0.001 |
| IGLV3-25 | 5.60 | <0.001 |
| IGHV1-24 | 6.25 | <0.001 |
| TNFRSF10A | 2.25 | <0.001 |
| IGLV1-47 | 5.04 | <0.001 |
| MMP9 | 6.54 | <0.001 |
| PPP3CA | 1.50 | <0.001 |
| IGHV1-2 | 5.08 | <0.001 |
| IGHV3-33 | 4.95 | <0.001 |
| TRAF2 | 1.34 | <0.001 |
| HBEGF | 2.58 | <0.001 |
| IL22RA1 | -3.25 | <0.001 |
| CARD11 | 4.32 | <0.001 |
| PPY | 4.83 | <0.001 |
| LIF | 2.90 | <0.001 |
| MMP12 | 9.26 | <0.001 |
| IGHV3-23 | 5.36 | <0.001 |
| S100A3 | 2.76 | <0.001 |
| IL17RB | -1.32 | <0.001 |
| PTGDS | 1.19 | <0.001 |
| C5AR1 | 1.71 | <0.001 |
| FABP3 | 3.01 | <0.001 |
| LYN | 1.41 | <0.001 |
| CRIM1 | 1.26 | <0.001 |
| MSR1 | 2.76 | <0.001 |
| CLCF1 | 1.35 | <0.001 |
| IGLV3-1 | 5.41 | <0.001 |
| IGKV1-17 | 5.58 | <0.001 |
| TGFA | 1.69 | <0.001 |
| PPBP | 4.00 | <0.001 |
| TAPBPL | 1.12 | <0.001 |
| IGKV1-33 | 5.72 | <0.001 |
| SHC2 | -2.68 | <0.001 |
| GRP | 3.09 | <0.001 |
| AGT | 1.79 | <0.001 |
| PROCR | 1.75 | <0.001 |
| CSF1R | 1.83 | <0.001 |
| INSR | -1.73 | <0.001 |
| SDC3 | 1.69 | <0.001 |
| IFNAR2 | 1.38 | <0.001 |
| SEMA3C | 4.78 | <0.001 |
| FPR1 | 2.08 | <0.001 |
| HMOX1 | 2.36 | <0.001 |
| S100B | 1.82 | <0.001 |
| DUOX1 | 2.42 | <0.001 |
| MX2 | 2.21 | <0.001 |
| IGLV2-23 | 5.31 | <0.001 |
| IGHV5-51 | 5.80 | <0.001 |
| HCST | 2.52 | <0.001 |
| PDGFA | -1.32 | <0.001 |
| IL1R2 | 3.64 | <0.001 |
| CHGB | 1.25 | <0.001 |
| TAP2 | 1.62 | <0.001 |
| ADM | 1.33 | <0.001 |
| IGKV4-1 | 5.10 | <0.001 |
| IL1RL1 | -1.31 | <0.001 |
| S100A2 | 7.37 | <0.001 |
| VCAM1 | 1.35 | <0.001 |
| IGLV2-11 | 4.47 | <0.001 |
| FGFRL1 | -1.78 | <0.001 |
| TRIM22 | 1.27 | <0.001 |
| OASL | 5.51 | <0.001 |
| IGKV2-28 | 5.06 | <0.001 |
| CCL5 | 3.40 | <0.001 |
| DKK1 | 6.46 | <0.001 |
| CD4 | 2.41 | <0.001 |
| MUC4 | 5.35 | <0.001 |
| SCTR | -1.72 | <0.001 |
| CASP3 | 1.11 | <0.001 |
| EDN1 | 1.23 | <0.001 |
| NFKBIE | 1.81 | <0.001 |
| PPARG | 3.67 | <0.001 |
| LCK | 3.89 | <0.001 |
| IGKV3-15 | 5.87 | <0.001 |
| BMP4 | 3.59 | <0.001 |
| TNFSF11 | 5.31 | <0.001 |
| INHBA | 6.29 | <0.001 |
| ANGPTL2 | 2.88 | <0.001 |
| ICAM2 | 1.24 | <0.001 |
| IRF1 | 1.54 | <0.001 |
| TNFRSF11B | 3.83 | <0.001 |
| FYN | 1.48 | <0.001 |
| PSMD14 | 1.30 | <0.001 |
| PNP | 1.86 | <0.001 |
| LIMS1 | 1.73 | <0.001 |
| DES | 1.15 | <0.001 |
| OLR1 | 3.53 | <0.001 |
| MET | 2.48 | <0.001 |
| IGKV1-5 | 5.45 | <0.001 |
| IGLV1-40 | 5.11 | <0.001 |
| ICAM1 | 2.17 | <0.001 |
| IGHV3-30 | 5.74 | <0.001 |
| IGHG3 | 7.07 | <0.001 |
| PDGFC | 1.38 | <0.001 |
| IGLV3-19 | 5.08 | <0.001 |
| IGLV1-51 | 4.51 | <0.001 |
| IL18 | 1.99 | <0.001 |
| PTAFR | 3.05 | <0.001 |
| CX3CL1 | 1.54 | <0.001 |
| TNC | 3.93 | <0.001 |
| TGFB2 | 2.18 | <0.001 |
| F2R | 3.45 | <0.001 |
| TRBV30 | 4.17 | <0.001 |
| CD14 | 1.91 | <0.001 |
| BMP1 | 1.36 | <0.001 |
| CXCL6 | 1.70 | <0.001 |
| LTBP2 | 2.34 | <0.001 |
| NRP2 | 4.04 | <0.001 |
| RARG | 1.86 | <0.001 |
| IL13RA1 | 1.40 | <0.001 |
| PSMD1 | 1.40 | <0.001 |
| IGLV2-14 | 4.67 | <0.001 |
| RARA | 1.48 | <0.001 |
| BID | 1.73 | <0.001 |
| PTPN6 | 1.09 | <0.001 |
| STAT1 | 1.69 | <0.001 |
| CSK | 1.37 | <0.001 |
| CLEC11A | 2.19 | <0.001 |
| IGHG2 | 5.81 | <0.001 |
| CCL17 | 6.61 | <0.001 |
| TNFRSF6B | 4.35 | <0.001 |
| RAC2 | 3.66 | <0.001 |
| SLC40A1 | 1.41 | <0.001 |
| IRF7 | 1.25 | <0.001 |
| FGFR1 | -1.77 | <0.001 |
| FCGR3A | 3.10 | <0.001 |
| GMFG | 2.37 | <0.001 |
| SYTL1 | -2.19 | <0.001 |
| IGKV1D-39 | 5.89 | <0.001 |
| PSMD6 | -2.62 | <0.001 |
| TGFB1 | 1.61 | <0.001 |
| LAP3 | 1.43 | <0.001 |
| CMTM6 | 1.69 | <0.001 |
| CD3D | 3.84 | <0.001 |
| CTSS | 3.45 | <0.001 |
| PLXNB1 | -1.27 | <0.001 |
| TNFSF10 | 1.68 | <0.001 |
| CMTM7 | 1.40 | <0.001 |
| SOCS3 | 1.21 | <0.001 |
| CST4 | 8.12 | <0.001 |
| IGKV3-20 | 5.45 | <0.001 |
| PLXND1 | 1.09 | <0.001 |
| TAP1 | 2.08 | <0.001 |
| ISG20 | 2.48 | <0.001 |
| ITGAV | 1.44 | <0.001 |
| HLA-DMB | 2.63 | <0.001 |
| MARCO | 4.45 | <0.001 |
| CRABP2 | 5.35 | <0.001 |
| PML | 1.58 | <0.001 |
| IL1RN | 5.28 | <0.001 |
| CMTM3 | 3.33 | <0.001 |
| IGHG4 | 8.39 | <0.001 |
| SCGB3A1 | 1.78 | <0.001 |
| GREM1 | 4.30 | <0.001 |
| PSMC4 | 1.24 | <0.001 |
| CXCL14 | 3.82 | <0.001 |
| AREG | 4.13 | <0.001 |
| GBP2 | 2.85 | <0.001 |
| FAM3B | -1.71 | <0.001 |
| CXCR4 | 3.65 | <0.001 |
| MX1 | 2.65 | <0.001 |
| HLA-H | 3.05 | <0.001 |
| LTBP1 | 3.40 | <0.001 |
| CXCL8 | 2.45 | <0.001 |
| PDGFRB | 1.80 | <0.001 |
| OAS1 | 3.96 | <0.001 |
| ITGB2 | 2.88 | <0.001 |
| DDX17 | -1.45 | <0.001 |
| CKLF | 2.79 | <0.001 |
| LTB | 3.90 | <0.001 |
| PSMB8 | 2.17 | <0.001 |
| UNC93B1 | 1.89 | <0.001 |
| CXCL16 | 2.25 | <0.001 |
| LGMN | 1.88 | <0.001 |
| IGKV3-11 | 5.22 | <0.001 |
| HLA-DMA | 2.16 | <0.001 |
| IFNGR2 | 1.98 | <0.001 |
| FAM3D | 6.42 | <0.001 |
| CCL20 | 4.99 | <0.001 |
| GPI | 1.25 | <0.001 |
| SAA2 | 1.73 | <0.001 |
| DUOX2 | 5.68 | <0.001 |
| PLAU | 4.42 | <0.001 |
| FCER1G | 2.32 | <0.001 |
| TAPBP | 1.27 | <0.001 |
| HLA-F | 2.61 | <0.001 |
| LRP1 | 2.07 | <0.001 |
| AHNAK | 1.62 | <0.001 |
| THBS1 | 2.04 | <0.001 |
| IGHM | 4.53 | <0.001 |
| TNFRSF21 | 2.79 | <0.001 |
| RBP1 | -1.74 | <0.001 |
| CCL18 | 7.75 | <0.001 |
| TYMP | 1.92 | <0.001 |
| IGHA2 | 3.51 | <0.001 |
| PPP4C | 1.26 | <0.001 |
| PSME1 | 1.22 | <0.001 |
| CXCL5 | 7.28 | <0.001 |
| ISG15 | 4.18 | <0.001 |
| SDC1 | 3.56 | <0.001 |
| MAPK3 | 1.78 | <0.001 |
| TINAGL1 | 1.75 | <0.001 |
| CDC42 | 1.28 | <0.001 |
| HDGF | 1.32 | <0.001 |
| PLAUR | 4.57 | <0.001 |
| IGLC1 | 5.30 | <0.001 |
| SOD1 | 1.37 | <0.001 |
| HLA-DQA1 | 4.29 | <0.001 |
| SDC4 | 1.70 | <0.001 |
| MUC5AC | 5.85 | <0.001 |
| HLA-DPA1 | 3.26 | <0.001 |
| BST2 | 3.27 | <0.001 |
| MDK | 3.90 | <0.001 |
| IL2RG | 5.48 | <0.001 |
| TYROBP | 2.77 | <0.001 |
| IFITM1 | 2.06 | <0.001 |
| A2M | 1.48 | <0.001 |
| IGLC3 | 4.86 | <0.001 |
| AZGP1 | -2.56 | <0.001 |
| HLA-DQB1 | 3.72 | <0.001 |
| IGHG1 | 6.47 | <0.001 |
| HLA-DRB5 | 3.54 | <0.001 |
| IFI30 | 2.36 | <0.001 |
| ZYX | 1.15 | <0.001 |
| PRDX1 | 1.86 | <0.001 |
| RAC1 | 1.66 | <0.001 |
| S100A13 | 1.75 | <0.001 |
| HLA-DPB1 | 3.31 | <0.001 |
| IGLC2 | 4.90 | <0.001 |
| SAA1 | 1.74 | <0.001 |
| MIA | 6.80 | <0.001 |
| HLA-E | 1.59 | <0.001 |
| REG1A | -3.88 | <0.001 |
| TRBC2 | 4.43 | <0.001 |
| HSPA8 | -1.00 | <0.001 |
| DEFB1 | -1.74 | <0.001 |
| PFN1 | 1.57 | <0.001 |
| GRN | 2.22 | <0.001 |
| CLDN4 | 1.79 | <0.001 |
| CTSE | 7.26 | <0.001 |
| SPP1 | 1.52 | <0.001 |
| S100A14 | 4.26 | <0.001 |
| HLA-DRB1 | 3.49 | <0.001 |
| TPM2 | 1.90 | <0.001 |
| SLPI | 4.33 | <0.001 |
| CALR | 1.12 | <0.001 |
| HLA-C | 1.93 | <0.001 |
| S100A16 | 3.68 | <0.001 |
| IGHA1 | 3.91 | <0.001 |
| HLA-DRA | 3.79 | <0.001 |
| SERPINA3 | -2.59 | <0.001 |
| PSAP | 1.11 | <0.001 |
| WFDC2 | 3.90 | <0.001 |
| VIM | 1.38 | <0.001 |
| CTSB | 2.34 | <0.001 |
| S100A10 | 3.30 | <0.001 |
| IGKC | 5.15 | <0.001 |
| HLA-B | 2.57 | <0.001 |
| HLA-A | 3.04 | <0.001 |
| LYZ | 4.86 | <0.001 |
| LCN2 | 4.58 | <0.001 |
| S100P | 8.39 | <0.001 |
| S100A11 | 3.38 | <0.001 |
| CD74 | 3.18 | <0.001 |
| TMSB4X | 1.94 | <0.001 |
| TMSB10 | 3.09 | <0.001 |
| B2M | 2.33 | <0.001 |
| S100A6 | 5.49 | <0.001 |

TableS3 prognosis-related IRGs of PDAC by Kaplan-Meier analysis in TCGA database

| **ID** | **P value** |
| --- | --- |
| IGLV7-46 | <0.001 |
| FYN | <0.001 |
| GH1 | <0.001 |
| IL17B | 0.002591 |
| LCN10 | 0.003259 |
| TRBV2 | 0.004331 |
| S100A16 | 0.004635 |
| XCR1 | 0.004665 |
| CCL22 | 0.004858 |
| CCL23 | 0.004929 |
| ZAP70 | 0.006171 |
| TNFRSF13B | 0.006272 |
| PSMD6 | 0.006589 |
| TNF | 0.006959 |
| CTSG | 0.007378 |
| IL9R | 0.007425 |
| CD1D | 0.007659 |
| IL13RA2 | 0.007774 |
| CRHR2 | 0.008262 |
| CXCR4 | 0.008489 |
| TRAV13-1 | 0.009037 |
| TRBV9 | 0.009423 |
| PLA2G2A | 0.009513 |
| LYZ | 0.010018 |
| NPPC | 0.011965 |
| PRLR | 0.012172 |
| TAC1 | 0.013335 |
| TRAV8-4 | 0.014543 |
| PPP3CA | 0.014896 |
| CRLF2 | 0.01517 |
| TRGC1 | 0.015732 |
| PTPN6 | 0.017294 |
| EBI3 | 0.017315 |
| PRKCB | 0.018269 |
| CMTM6 | 0.020036 |
| CMA1 | 0.020789 |
| CD22 | 0.020891 |
| IGLV10-54 | 0.020943 |
| CLEC11A | 0.021798 |
| CCR7 | 0.022119 |
| MET | 0.022753 |
| BTK | 0.023712 |
| IL16 | 0.024698 |
| IGLC6 | 0.024924 |
| CCL19 | 0.025002 |
| TRGC2 | 0.02511 |
| RFX5 | 0.025212 |
| SEMA3C | 0.025463 |
| NFATC1 | 0.025849 |
| LCP2 | 0.02624 |
| TRBV12-4 | 0.026934 |
| CXCL3 | 0.027414 |
| TRAV8-2 | 0.027601 |
| S100Z | 0.027823 |
| DMBT1 | 0.028039 |
| PIK3R5 | 0.0285 |
| VAV1 | 0.028553 |
| CCR8 | 0.02856 |
| IL24 | 0.029166 |
| ITGAL | 0.029442 |
| IGLV2-8 | 0.029711 |
| TNFRSF4 | 0.029797 |
| SLC10A2 | 0.02985 |
| ERAP2 | 0.030512 |
| TRBV3-1 | 0.031354 |
| CSF2RB | 0.031485 |
| CR2 | 0.033916 |
| TNFRSF13C | 0.034343 |
| ITK | 0.034532 |
| TRBV6-6 | 0.035735 |
| S100B | 0.035868 |
| CCR9 | 0.036215 |
| CSK | 0.03695 |
| CAMP | 0.037081 |
| LCN6 | 0.037211 |
| TNFSF14 | 0.037621 |
| SCG2 | 0.038009 |
| CCR6 | 0.03882 |
| IGLC3 | 0.038855 |
| FLT3LG | 0.039597 |
| CCR4 | 0.04026 |
| CGB7 | 0.040303 |
| RAC1 | 0.040615 |
| OGN | 0.04074 |
| CRABP1 | 0.042702 |
| IGKV1D-17 | 0.043222 |
| IGLV3-25 | 0.043266 |
| RORA | 0.044096 |
| ANGPTL5 | 0.045166 |
| IGLV2-18 | 0.045777 |
| UCN3 | 0.045957 |
| ZYX | 0.048249 |
| IL1RL2 | 0.048282 |
| TRAV12-2 | 0.049578 |
| IGHM | 0.04962 |

TableS4 prognosis-related IRGs of PDAC by Univariable Cox regression analysis in TCGA database

| **ID** | **HR** | **HR.95L** | **HR.95H** | **P value** |
| --- | --- | --- | --- | --- |
| IL20RB | 1.562 | 1.319 | 1.851 | <0.001 |
| MET | 1.507 | 1.251 | 1.815 | <0.002 |
| CGB2 | 1.356 | 1.130 | 1.629 | 0.001 |
| PLAU | 1.285 | 1.099 | 1.501 | 0.002 |
| S100A2 | 1.345 | 1.115 | 1.622 | 0.002 |
| GH1 | 0.582 | 0.410 | 0.827 | 0.003 |
| IDO1 | 1.340 | 1.093 | 1.643 | 0.005 |
| FGF2 | 1.260 | 1.063 | 1.493 | 0.008 |
| NPPA | 0.673 | 0.501 | 0.904 | 0.009 |
| EREG | 1.259 | 1.059 | 1.497 | 0.009 |
| PPP3CA | 1.271 | 1.061 | 1.522 | 0.009 |
| ESR2 | 0.690 | 0.521 | 0.914 | 0.010 |
| CKLF | 1.359 | 1.076 | 1.717 | 0.010 |
| CCR10 | 0.709 | 0.544 | 0.925 | 0.011 |
| TNFSF10 | 1.207 | 1.044 | 1.395 | 0.011 |
| CRHR2 | 0.656 | 0.471 | 0.915 | 0.013 |
| IL17B | 0.550 | 0.343 | 0.882 | 0.013 |
| ERAP2 | 1.292 | 1.055 | 1.581 | 0.013 |
| CYSLTR1 | 1.333 | 1.062 | 1.675 | 0.013 |
| DTL | 1.307 | 1.057 | 1.615 | 0.013 |
| PIK3R5 | 0.704 | 0.533 | 0.930 | 0.014 |
| CD1D | 0.708 | 0.538 | 0.932 | 0.014 |
| FYN | 0.700 | 0.526 | 0.931 | 0.014 |
| IL9R | 0.704 | 0.530 | 0.934 | 0.015 |
| TNFRSF21 | 1.312 | 1.054 | 1.634 | 0.015 |
| CMTM1 | 1.264 | 1.041 | 1.535 | 0.018 |
| IKBKE | 0.694 | 0.511 | 0.942 | 0.019 |
| IL1RAP | 1.242 | 1.036 | 1.489 | 0.019 |
| INHBA | 1.273 | 1.039 | 1.561 | 0.020 |
| CMTM7 | 1.223 | 1.032 | 1.449 | 0.020 |
| TNFRSF4 | 0.714 | 0.537 | 0.949 | 0.020 |
| TRBV2 | 0.625 | 0.420 | 0.930 | 0.020 |
| OASL | 1.266 | 1.035 | 1.548 | 0.022 |
| IL1R2 | 1.235 | 1.031 | 1.479 | 0.022 |
| IGLV7-46 | 0.699 | 0.514 | 0.951 | 0.023 |
| TRBV6-1 | 0.633 | 0.425 | 0.942 | 0.024 |
| TRGC2 | 0.734 | 0.560 | 0.961 | 0.025 |
| TRAV8-4 | 0.625 | 0.415 | 0.942 | 0.025 |
| APLNR | 0.758 | 0.594 | 0.966 | 0.025 |
| IL16 | 0.683 | 0.489 | 0.954 | 0.025 |
| IL22RA1 | 1.258 | 1.027 | 1.542 | 0.027 |
| RSAD2 | 1.261 | 1.026 | 1.551 | 0.028 |
| CRABP2 | 1.234 | 1.022 | 1.490 | 0.028 |
| FLT3LG | 0.729 | 0.549 | 0.968 | 0.029 |
| AHNAK | 1.261 | 1.024 | 1.553 | 0.029 |
| ALB | 1.236 | 1.021 | 1.496 | 0.029 |
| BIRC5 | 1.207 | 1.019 | 1.429 | 0.030 |
| SHC2 | 0.783 | 0.628 | 0.977 | 0.031 |
| SEMA3A | 1.255 | 1.022 | 1.541 | 0.031 |
| IL11 | 1.194 | 1.017 | 1.403 | 0.031 |
| PRLR | 0.772 | 0.610 | 0.976 | 0.031 |
| TRAV8-6 | 0.679 | 0.477 | 0.967 | 0.032 |
| UCN2 | 1.227 | 1.017 | 1.481 | 0.033 |
| PSMD6 | 1.245 | 1.017 | 1.523 | 0.033 |
| IL10RA | 0.741 | 0.562 | 0.977 | 0.034 |
| TAC1 | 0.707 | 0.511 | 0.978 | 0.036 |
| TRAV8-2 | 0.712 | 0.518 | 0.978 | 0.036 |
| ITGAL | 0.664 | 0.452 | 0.976 | 0.037 |
| TRBV4-2 | 0.742 | 0.560 | 0.982 | 0.037 |
| VAV1 | 0.750 | 0.572 | 0.985 | 0.038 |
| SEMA3C | 1.222 | 1.011 | 1.476 | 0.038 |
| CD247 | 0.661 | 0.445 | 0.982 | 0.040 |
| TAP2 | 1.228 | 1.009 | 1.495 | 0.041 |
| CCL22 | 0.732 | 0.544 | 0.987 | 0.041 |
| CCR9 | 0.734 | 0.546 | 0.987 | 0.041 |
| GRAP2 | 0.720 | 0.525 | 0.987 | 0.041 |
| IGHA2 | 0.797 | 0.641 | 0.991 | 0.041 |
| PTGS2 | 1.174 | 1.006 | 1.370 | 0.042 |
| TRBV5-6 | 0.635 | 0.410 | 0.984 | 0.042 |
| TRAC | 0.683 | 0.473 | 0.987 | 0.042 |
| IGHV3-49 | 0.795 | 0.635 | 0.994 | 0.045 |
| TIE1 | 0.798 | 0.639 | 0.995 | 0.045 |
| CRHR1 | 0.718 | 0.516 | 0.997 | 0.048 |

TableS5 prognosis-related IRGs of PDAC by Kaplan-Meier analysis in ICGC database

| **ID** | **P value** |
| --- | --- |
| SPP1 | <0.001 |
| RAC2 | <0.001 |
| TMSB10 | <0.001 |
| B2M | <0.001 |
| EREG | <0.001 |
| PTHLH | <0.001 |
| IGKV1D-16 | <0.001 |
| FCGR3A | <0.001 |
| IFITM1 | <0.001 |
| AREG | <0.001 |
| TNFRSF6B | 0.001 |
| STAT1 | 0.001 |
| APOBEC3G | 0.001 |
| HSPA6 | 0.001 |
| IL11 | 0.001 |
| CMTM1 | 0.002 |
| S100A3 | 0.002 |
| IL20RB | 0.002 |
| TYMP | 0.002 |
| PAEP | 0.002 |
| DKK1 | 0.002 |
| IGHV1-69 | 0.002 |
| SERPINA3 | 0.003 |
| ISG15 | 0.003 |
| SOD1 | 0.003 |
| VAV1 | 0.003 |
| IGLV10-54 | 0.004 |
| FABP2 | 0.004 |
| LYZ | 0.004 |
| SLIT3 | 0.004 |
| CD70 | 0.004 |
| ADM | 0.005 |
| CCL8 | 0.006 |
| OGN | 0.006 |
| HLA-G | 0.006 |
| CGB7 | 0.007 |
| PLXNA2 | 0.007 |
| TGFA | 0.008 |
| NTF4 | 0.008 |
| CD86 | 0.008 |
| IGHA2 | 0.009 |
| RORC | 0.009 |
| IGHG4 | 0.010 |
| IL1A | 0.010 |
| ROR2 | 0.010 |
| FGF11 | 0.011 |
| MMP9 | 0.011 |
| RAET1E | 0.011 |
| MET | 0.011 |
| IGKV3D-15 | 0.012 |
| IGLC1 | 0.012 |
| PI15 | 0.012 |
| IGHV4-34 | 0.015 |
| CMTM3 | 0.015 |
| PTX3 | 0.015 |
| IFI30 | 0.016 |
| IGKV3D-20 | 0.016 |
| PPP3CA | 0.016 |
| AQP9 | 0.017 |
| IGLV2-14 | 0.017 |
| IGLV7-46 | 0.018 |
| RBP2 | 0.018 |
| BID | 0.018 |
| A2M | 0.018 |
| SAA2 | 0.019 |
| CGB5 | 0.020 |
| RAET1G | 0.021 |
| DLL4 | 0.021 |
| REG3G | 0.022 |
| LIMS1 | 0.022 |
| IGHV3-23 | 0.023 |
| IGKV1D-39 | 0.023 |
| TCF4 | 0.023 |
| IGHA1 | 0.024 |
| IGKV1D-13 | 0.024 |
| IL17RD | 0.025 |
| PLXND1 | 0.025 |
| EPOR | 0.025 |
| IGHV3-73 | 0.025 |
| SEMA3A | 0.026 |
| IL2RA | 0.026 |
| PSMD14 | 0.026 |
| IGLV2-18 | 0.027 |
| MX1 | 0.027 |
| IGHG1 | 0.027 |
| PIK3CG | 0.027 |
| IGHV3-53 | 0.028 |
| S100A2 | 0.028 |
| CMTM8 | 0.028 |
| AZGP1 | 0.028 |
| OLR1 | 0.029 |
| DUOX1 | 0.029 |
| IGLV3-19 | 0.029 |
| NOD2 | 0.030 |
| IGHV2-70 | 0.031 |
| PRLR | 0.032 |
| LGR5 | 0.032 |
| CD72 | 0.033 |
| FYN | 0.033 |
| SEMA7A | 0.033 |
| FPR1 | 0.033 |
| IRF7 | 0.034 |
| CXCL6 | 0.035 |
| MICB | 0.036 |
| FCER1G | 0.036 |
| FGF19 | 0.036 |
| TNFRSF10C | 0.038 |
| IGHV3-20 | 0.039 |
| HCST | 0.040 |
| SECTM1 | 0.040 |
| IGHV4-31 | 0.040 |
| IGKV1-9 | 0.040 |
| FGF18 | 0.040 |
| IGLV1-44 | 0.040 |
| BST2 | 0.041 |
| TNFRSF18 | 0.043 |
| CXCL10 | 0.044 |
| CCL13 | 0.044 |
| CMTM7 | 0.045 |
| LCP2 | 0.047 |
| C5AR1 | 0.048 |
| CCRL2 | 0.048 |
| HLA-H | 0.048 |
| CRIM1 | 0.049 |
| CTLA4 | 0.049 |

TableS6 prognosis-related IRGs of PDAC by Univariable Cox regression analysis in ICGC database

| ID | HR | HR.95L | HR.95H | P value |
| --- | --- | --- | --- | --- |
| RAC2 | 1.846 | 1.406 | 2.423 | <0.001 |
| SPP1 | 1.868 | 1.403 | 2.486 | <0.001 |
| PAEP | 1.694 | 1.311 | 2.189 | <0.001 |
| DKK1 | 1.750 | 1.314 | 2.330 | <0.001 |
| EREG | 1.659 | 1.276 | 2.156 | <0.001 |
| HSPA6 | 1.517 | 1.217 | 1.892 | <0.001 |
| RORC | 0.633 | 0.496 | 0.809 | <0.001 |
| TYMP | 1.543 | 1.216 | 1.957 | <0.001 |
| HLA-G | 1.627 | 1.245 | 2.125 | <0.001 |
| IL1A | 1.602 | 1.236 | 2.078 | <0.001 |
| CD70 | 1.662 | 1.256 | 2.200 | <0.001 |
| TMSB10 | 1.585 | 1.219 | 2.061 | 0.001 |
| S100A2 | 1.556 | 1.207 | 2.006 | 0.001 |
| LYZ | 0.640 | 0.491 | 0.834 | 0.001 |
| IFITM1 | 1.552 | 1.194 | 2.016 | 0.001 |
| PTHLH | 1.533 | 1.187 | 1.980 | 0.001 |
| ADM | 1.579 | 1.196 | 2.084 | 0.001 |
| TGFB1 | 1.554 | 1.187 | 2.034 | 0.001 |
| APOBEC3G | 1.630 | 1.201 | 2.213 | 0.002 |
| CMTM3 | 1.537 | 1.171 | 2.017 | 0.002 |
| FGF11 | 1.483 | 1.156 | 1.903 | 0.002 |
| PLAU | 1.513 | 1.163 | 1.967 | 0.002 |
| PRLR | 0.688 | 0.542 | 0.874 | 0.002 |
| PTX3 | 1.518 | 1.161 | 1.984 | 0.002 |
| SEMA3A | 1.583 | 1.179 | 2.125 | 0.002 |
| RAET1E | 1.481 | 1.146 | 1.914 | 0.003 |
| S100A3 | 1.429 | 1.131 | 1.806 | 0.003 |
| LIMS1 | 1.372 | 1.115 | 1.688 | 0.003 |
| MET | 1.475 | 1.134 | 1.917 | 0.004 |
| MMP9 | 1.404 | 1.116 | 1.766 | 0.004 |
| ULBP2 | 1.478 | 1.135 | 1.924 | 0.004 |
| PLXNA2 | 0.678 | 0.521 | 0.882 | 0.004 |
| CXCL6 | 1.519 | 1.142 | 2.019 | 0.004 |
| FAM3D | 0.698 | 0.546 | 0.892 | 0.004 |
| NTF4 | 1.476 | 1.131 | 1.926 | 0.004 |
| MICB | 1.442 | 1.122 | 1.853 | 0.004 |
| FGF19 | 1.478 | 1.129 | 1.934 | 0.004 |
| TGFA | 1.509 | 1.135 | 2.006 | 0.005 |
| IGLV2-18 | 0.701 | 0.547 | 0.898 | 0.005 |
| TGFB2 | 1.427 | 1.112 | 1.831 | 0.005 |
| VAV1 | 1.482 | 1.122 | 1.957 | 0.006 |
| PTGER2 | 0.733 | 0.588 | 0.914 | 0.006 |
| SLIT3 | 0.682 | 0.519 | 0.896 | 0.006 |
| ISG15 | 1.373 | 1.094 | 1.724 | 0.006 |
| IL18 | 1.474 | 1.115 | 1.949 | 0.006 |
| SERPINA3 | 1.475 | 1.112 | 1.957 | 0.007 |
| SOD1 | 0.689 | 0.525 | 0.903 | 0.007 |
| IL20RB | 1.398 | 1.095 | 1.786 | 0.007 |
| TNFRSF6B | 1.497 | 1.114 | 2.012 | 0.007 |
| STC1 | 1.426 | 1.098 | 1.853 | 0.008 |
| IGHV3-53 | 0.715 | 0.557 | 0.917 | 0.008 |
| IGHA2 | 0.713 | 0.555 | 0.916 | 0.008 |
| TDGF1 | 0.718 | 0.561 | 0.918 | 0.008 |
| EPOR | 1.386 | 1.087 | 1.766 | 0.008 |
| APLNR | 0.737 | 0.587 | 0.925 | 0.009 |
| ICAM1 | 1.403 | 1.089 | 1.806 | 0.009 |
| CCR1 | 1.400 | 1.084 | 1.808 | 0.010 |
| CCL24 | 0.728 | 0.572 | 0.926 | 0.010 |
| PI15 | 1.455 | 1.094 | 1.935 | 0.010 |
| IGKV1D-13 | 0.703 | 0.537 | 0.919 | 0.010 |
| FCGR3A | 1.357 | 1.076 | 1.713 | 0.010 |
| AZGP1 | 0.723 | 0.564 | 0.925 | 0.010 |
| STAT1 | 1.363 | 1.076 | 1.727 | 0.010 |
| NOD2 | 1.401 | 1.082 | 1.815 | 0.011 |
| BST2 | 1.434 | 1.086 | 1.893 | 0.011 |
| CTSE | 0.735 | 0.579 | 0.932 | 0.011 |
| IRF7 | 1.383 | 1.077 | 1.777 | 0.011 |
| FGF13 | 0.728 | 0.567 | 0.934 | 0.013 |
| AREG | 1.363 | 1.069 | 1.739 | 0.013 |
| SEMA7A | 1.338 | 1.064 | 1.684 | 0.013 |
| PSMD14 | 1.372 | 1.069 | 1.760 | 0.013 |
| CCRL2 | 0.727 | 0.565 | 0.935 | 0.013 |
| MMP12 | 1.377 | 1.069 | 1.774 | 0.013 |
| IGHV3-64 | 0.732 | 0.572 | 0.937 | 0.013 |
| SLC40A1 | 0.707 | 0.537 | 0.930 | 0.013 |
| CRIM1 | 0.736 | 0.577 | 0.941 | 0.014 |
| LGR5 | 0.724 | 0.559 | 0.938 | 0.014 |
| ALB | 1.371 | 1.063 | 1.767 | 0.015 |
| DUOX1 | 1.410 | 1.068 | 1.861 | 0.015 |
| IL7 | 0.748 | 0.590 | 0.948 | 0.016 |
| TNFRSF4 | 1.375 | 1.059 | 1.784 | 0.017 |
| CMTM8 | 0.709 | 0.535 | 0.940 | 0.017 |
| RBP2 | 0.735 | 0.571 | 0.947 | 0.017 |
| B2M | 1.372 | 1.058 | 1.781 | 0.017 |
| TNFRSF18 | 1.341 | 1.053 | 1.708 | 0.017 |
| CGB7 | 1.395 | 1.058 | 1.840 | 0.018 |
| CCL8 | 1.396 | 1.057 | 1.844 | 0.019 |
| IGHV3-20 | 0.734 | 0.566 | 0.951 | 0.020 |
| ICAM2 | 1.359 | 1.050 | 1.758 | 0.020 |
| RAET1G | 1.393 | 1.051 | 1.846 | 0.021 |
| IGKV3D-20 | 0.746 | 0.581 | 0.957 | 0.021 |
| IGHV3-72 | 0.747 | 0.581 | 0.960 | 0.023 |
| IGLV4-69 | 0.742 | 0.574 | 0.959 | 0.023 |
| IGKV4-1 | 0.741 | 0.572 | 0.960 | 0.023 |
| BMP1 | 1.319 | 1.038 | 1.676 | 0.024 |
| BIRC5 | 1.339 | 1.040 | 1.724 | 0.024 |
| CMTM1 | 1.321 | 1.037 | 1.682 | 0.024 |
| IGKV3D-15 | 0.768 | 0.611 | 0.966 | 0.024 |
| BID | 1.314 | 1.036 | 1.665 | 0.024 |
| AQP9 | 1.300 | 1.035 | 1.634 | 0.024 |
| SECTM1 | 1.351 | 1.037 | 1.759 | 0.026 |
| CGB5 | 1.329 | 1.033 | 1.709 | 0.027 |
| ROBO2 | 0.758 | 0.593 | 0.969 | 0.027 |
| IL33 | 0.773 | 0.615 | 0.972 | 0.028 |
| IGHV3-21 | 0.753 | 0.585 | 0.969 | 0.028 |
| HMOX1 | 1.287 | 1.025 | 1.616 | 0.030 |
| TNFRSF11A | 0.746 | 0.572 | 0.972 | 0.030 |
| IGHV3-23 | 0.756 | 0.587 | 0.973 | 0.030 |
| NR1I2 | 0.763 | 0.598 | 0.974 | 0.030 |
| FGF18 | 0.742 | 0.566 | 0.972 | 0.031 |
| IGLV2-14 | 0.762 | 0.595 | 0.975 | 0.031 |
| IGHV3-73 | 0.766 | 0.601 | 0.975 | 0.031 |
| CXCL3 | 0.760 | 0.592 | 0.976 | 0.032 |
| CTSS | 0.766 | 0.600 | 0.977 | 0.032 |
| CD28 | 1.311 | 1.022 | 1.682 | 0.033 |
| IGHV5-51 | 0.752 | 0.578 | 0.977 | 0.033 |
| CCL13 | 1.304 | 1.021 | 1.665 | 0.033 |
| IGKV1D-39 | 0.754 | 0.581 | 0.978 | 0.033 |
| CD86 | 1.293 | 1.020 | 1.640 | 0.034 |
| IGHG4 | 0.748 | 0.572 | 0.979 | 0.034 |
| IGHV3-7 | 0.764 | 0.594 | 0.981 | 0.035 |
| MSR1 | 1.277 | 1.016 | 1.605 | 0.036 |
| FABP2 | 0.766 | 0.595 | 0.986 | 0.038 |
| IGHG2 | 0.765 | 0.593 | 0.987 | 0.039 |
| CD22 | 1.345 | 1.015 | 1.784 | 0.039 |
| IGHV3-49 | 0.761 | 0.586 | 0.987 | 0.040 |
| IL11 | 1.284 | 1.012 | 1.629 | 0.040 |
| CCL3 | 1.278 | 1.011 | 1.616 | 0.040 |
| IGLV1-40 | 0.761 | 0.586 | 0.989 | 0.041 |
| IGLV7-46 | 0.771 | 0.601 | 0.989 | 0.041 |
| IGKV1D-16 | 0.777 | 0.611 | 0.990 | 0.041 |
| IGHA1 | 0.774 | 0.605 | 0.990 | 0.041 |
| IGHV1-46 | 0.765 | 0.590 | 0.990 | 0.042 |
| VEGFC | 1.339 | 1.009 | 1.778 | 0.043 |
| IGLV5-45 | 0.765 | 0.590 | 0.992 | 0.043 |
| IGKV3-20 | 0.770 | 0.597 | 0.992 | 0.044 |
| IGKV1-27 | 0.773 | 0.602 | 0.994 | 0.044 |
| TRIM22 | 1.313 | 1.006 | 1.713 | 0.045 |
| CD72 | 1.276 | 1.004 | 1.621 | 0.046 |
| IGHV4-4 | 0.761 | 0.581 | 0.996 | 0.047 |
| MX1 | 1.300 | 1.003 | 1.685 | 0.048 |
| RSAD2 | 1.298 | 1.002 | 1.681 | 0.048 |
| IGKC | 0.777 | 0.605 | 0.999 | 0.049 |
| CXCL16 | 0.785 | 0.617 | 0.999 | 0.049 |
| IGHV3-15 | 0.782 | 0.611 | 1.000 | 0.050 |

TableS7 Different expression TFs between PDAC and normal pancreas

| TF | log2FoldChange | P adj |
| --- | --- | --- |
| EPO | -4.55 | <0.001 |
| XBP1 | -2.66 | <0.001 |
| OGT | -2.35 | <0.001 |
| SIX5 | -2.27 | <0.001 |
| GATA4 | -2.11 | <0.001 |
| SCML2 | -2.08 | <0.001 |
| GATA2 | -1.96 | <0.001 |
| MEIS1 | -1.83 | <0.001 |
| FOXP2 | -1.70 | <0.001 |
| ELL2 | -1.67 | <0.001 |
| KLF11 | -1.35 | <0.001 |
| BCL6 | -1.35 | <0.001 |
| NFIC | -1.18 | <0.001 |
| SNAPC4 | -1.16 | <0.001 |
| RBL2 | -1.03 | <0.001 |
| JUN | -1.01 | <0.001 |
| H2AFX | 1.04 | <0.001 |
| RUNX1T1 | 1.09 | <0.001 |
| MITF | 1.12 | <0.001 |
| WWTR1 | 1.12 | <0.001 |
| RYBP | 1.12 | <0.001 |
| RFX5 | 1.12 | <0.001 |
| LIN9 | 1.13 | <0.001 |
| ERG | 1.14 | <0.001 |
| CEBPB | 1.15 | <0.001 |
| YAP1 | 1.21 | <0.001 |
| LMO2 | 1.24 | <0.001 |
| CTNNB1 | 1.26 | <0.001 |
| RFX2 | 1.30 | <0.001 |
| BRCA1 | 1.30 | <0.001 |
| TP73 | 1.32 | <0.001 |
| SUMO1 | 1.41 | <0.001 |
| RBP2 | 1.41 | <0.001 |
| NFE2 | 1.41 | <0.001 |
| CIITA | 1.42 | <0.001 |
| SOX4 | 1.46 | <0.001 |
| IRF1 | 1.54 | <0.001 |
| ELF5 | 1.56 | <0.001 |
| MEF2C | 1.57 | <0.001 |
| BHLHE40 | 1.57 | <0.001 |
| LYL1 | 1.65 | <0.001 |
| HIF1A | 1.72 | <0.001 |
| MAF | 1.73 | <0.001 |
| LHX2 | 1.74 | <0.001 |
| E2F3 | 1.90 | <0.001 |
| MAFF | 1.95 | <0.001 |
| ETS1 | 1.96 | <0.001 |
| LMNA | 2.05 | <0.001 |
| HOXA9 | 2.17 | <0.001 |
| E2F7 | 2.40 | <0.001 |
| LMNB1 | 2.50 | <0.001 |
| IKZF1 | 2.58 | <0.001 |
| E2F1 | 2.67 | <0.001 |
| PRDM1 | 2.79 | <0.001 |
| ETV1 | 2.84 | <0.001 |
| VDR | 2.96 | <0.001 |
| RUNX1 | 3.03 | <0.001 |
| SPIB | 3.07 | <0.001 |
| IRF4 | 3.12 | <0.001 |
| KLF5 | 3.19 | <0.001 |
| FOXM1 | 3.23 | <0.001 |
| FOXA1 | 3.26 | <0.001 |
| PPARG | 3.67 | <0.001 |
| FOSL1 | 3.85 | <0.001 |
| MYB | 4.03 | <0.001 |
| FOXP3 | 4.22 | <0.001 |
| HOXB7 | 4.27 | <0.001 |
| HOXC9 | 4.47 | <0.001 |
| GATA3 | 4.67 | <0.001 |
| LEF1 | 4.69 | <0.001 |
| BATF | 4.77 | <0.001 |
| TFAP2A | 4.91 | <0.001 |
| HOXC11 | 7.04 | <0.001 |

TableS8 Relationships between the proportions of TIICs and the clinicopathological factors in papillary pancreatic adenocarcinoma: t(P)

| **gene** | **Age**  **(<60/≥65)** | **Gender**  **(female /male)** | **Grade**  **(G1-G2/**  **G3-G4)** | **Stage**  **(stageⅠ-Ⅱ/ stageⅢ-Ⅳ)** | **T**  **(T1-T2/**  **T3-T4)** | **N**  **(N0/ N1)** |
| --- | --- | --- | --- | --- | --- | --- |
| **naive B cells** | 0.02(0.984) | 0.02(0.984) | 1.611(0.116) | 0.048(0.963) | -0.239(0.814) | 0.978(0.334) |
| **memory B cells** | -0.668(0.507) | -0.668(0.507) | 0.08(0.936) | 1.7(0.139) | -0.292(0.774) | -0.354(0.726) |
| **Plasma cells** | -1.543(0.129) | -1.543(0.129) | 2.16(0.037) | -0.281(0.791) | -0.121(0.905) | 1.389(0.174) |
| **CD8+T cells** | -0.916(0.363) | -0.916(0.363) | 1.064(0.297) | 0.157(0.882) | 1.124(0.274) | -1.126(0.267) |
| **naive CD4 +T cells** | 0.965(0.340) | 0.965(0.340) | 1.293(0.201) | 1.292(0.201) | -0.427(0.671) | -0.611(0.543) |
| **memory resting CD4+T cells** | 1.324(0.190) | 1.324(0.190) | 0.334(0.741) | -0.45(0.674) | 0.313(0.758) | 0.588(0.561) |
| **memory activated CD4 +T cells** | -1.398(0.170) | -1.398(0.170) | 2.42(0.019) | 2.402(0.019) | 1.006(0.333) | 1.142(0.265) |
| **follicular helper T cells** | 0.125(0.901) | 0.125(0.901) | -0.946(0.354) | 0.256(0.809) | 0.181(0.859) | -0.084(0.934) |
| **Regulatory T cells (Tregs)** | 0.486(0.629) | 0.486(0.629) | 0.535(0.596) | 1.665(0.150) | 1.199(0.250) | 0.891(0.380) |
| **gamma delta T cells** | -1(0.325) | -1(0.325) | 1(0.322) | -1(0.374) | -1(0.321) | -1(0.322) |
| **resting NK cells** | 1.176(0.245) | 1.176(0.245) | -0.999(0.332) | 1.957(0.054) | -1.595(0.115) | -1.672(0.100) |
| **activated NK cells** | 0.534(0.595) | 0.534(0.595) | -1.367(0.185) | 0.387(0.716) | 0.856(0.404) | 1.106(0.278) |
| **Monocytes** | -0.059(0.953) | -0.059(0.953) | -1.037(0.313) | 1.461(0.197) | -0.547(0.590) | -0.518(0.606) |
| **M0 Macrophages** | 0.666(0.508) | 0.666(0.508) | -1.724(0.095) | 0.559(0.601) | -2.334(0.026) | -0.743(0.462) |
| **M1 Macrophages** | -0.677(0.500) | -0.677(0.500) | -0.493(0.628) | -0.528(0.621) | 1.753(0.097) | 0.364(0.717) |
| **M2 Macrophages** | 0.17(0.865) | 0.17(0.865) | -0.343(0.735) | -1.832(0.124) | -0.366(0.718) | -1.11(0.273) |
| **resting Dendritic cells** | 1.712(0.091) | 1.712(0.091) | 0.581(0.565) | 1.524(0.173) | 1.401(0.183) | 0.998(0.327) |
| **activated Dendritic cells** | 0.72(0.474) | 0.72(0.474) | 1.148(0.261) | 0.941(0.392) | 0.122(0.904) | 0.687(0.497) |
| **resting Mast cells** | -0.735(0.466) | -0.735(0.466) | 0.238(0.814) | -1.024(0.357) | 0.834(0.418) | -0.531(0.598) |
| **activated Mast cells** | -0.58(0.565) | -0.58(0.565) | -0.555(0.582) | 0.607(0.554) | -0.045(0.965) | -1.179(0.242) |
| **Eosinophils** | -0.797(0.431) | -0.797(0.431) | 0.39(0.698) | 1.242(0.218) | -0.161(0.873) | -1.244(0.219) |
